# Two decades of climate driving the dynamics of functional and taxonomic diversity of a tropical small mammal community in western Mexico

**DOI:** 10.1371/journal.pone.0189104

**Published:** 2017-12-11

**Authors:** Edgard David Mason-Romo, Ariel A. Farías, Gerardo Ceballos

**Affiliations:** 1 Laboratorio de Ecología y Conservación de Fauna Silvestre, Departamento de Ecología de la Biodiversidad, Instituto de Ecología, Universidad Nacional Autónoma de México, México City, México; 2 Centro Universitario Regional del Este (CURE), Maldonado, Punta del Este, Uruguay; Sichuan University, CHINA

## Abstract

Understanding the effects of global climate disruption on biodiversity is important to future conservation efforts. While taxonomic diversity is widely studied, functional diversity of plants, and recently animals, is receiving increasing attention. Most studies of mammals are short-term, focus on temperate habitats, and rely on traits described in the literature rather than generating traits from observations. Unlike previous studies, this long-term field study assessed the factors driving the functional and taxonomic diversity of small-mammal assemblages in dry tropical forests using both traits recorded from literature and a demographic database. We assessed the drivers (abundance and biomass, temperature and rainfall) of taxonomic richness and functional diversity for two rain-driven seasons in two adjacent but distinct forests—upland and lowland (arroyo or riparian) forests. Our analysis found that rainfall, both seasonal and atypical, was the primary factor driving functional and taxonomic diversity of small-mammal assemblages. Functional responses differed between the two types of forests, however, with effects being stronger in the harsher conditions of the upland forests than in the less severe conditions prevailing in the arroyo (riparian) forest. The latter also supports a richer, more diverse, and more stable small-mammal assemblage. These findings highlight the importance of climate to tropical biological diversity, as extreme climate events (hurricanes, droughts and floods) and disruption of rainfall patterns were shown to decrease biodiversity. They also support the need to preserve these habitats, as their high taxonomic diversity and functional redundancy makes them resilient against global climate disruption and local extreme events. Tropical dry forests constitute a potential reservoir for biodiversity and the ecosystem services they provide. Unfortunately, these forests are among the most endangered terrestrial ecosystems because of deforestation and the likely impacts of global climate disruption.

## Introduction

Understanding the drivers and consequences of biological diversity is a central objective for biologists and ecologists [1–3]. The current human-caused disruption in global climate has made this issue the more urgent, as projections include global increases in temperature, disruptions in rainfall patterns and a higher probability of extreme climate events [4–11], which may affect biodiversity [12]. In fact, climate change scenarios have already become dramatic reality [12–25], accelerating species loss and raising concerns about its effects on ecosystem processes and functioning, provision of ecosystem goods and services, and environmental sustainability [26–28]. The effects of species loss on ecosystem functioning constitute a complex issue. One way to analyze the consequences of species loss is to assess functional diversity. Functional diversity is the conjunction of components—species and their functional traits—that influence how ecosystems function[29–32], and it is measured as the variability in organisms’ traits related to ecosystem functioning. Drivers of functional diversity drivers are biotic (such as interactions among species), abiotic (such as environmental filtering), or stochastic (random processes, not ascribed to biotic or abiotic causes) [33–35]. Traits are defined as organisms’ characteristics that affect how they respond to or affect their environment; traits are highly relevant to organisms’ maintenance of the ecosystem services of the areas they inhabit (such as nutrient cycling in the soil by colonial prairie dogs, or habitat provision by aquatic dam-building beavers) [1,31,34,36–40]. Thus, analysis of functional diversity is based on the assumption that negative impacts on this biodiversity component will impair ecosystem processes [26,28].

Local species richness and intrinsic functional redundancy in the species pool (i.e., the overall similarity in functional traits among species in the regional assemblage [32]) determine how functional diversity responds to random changes in species number and composition (species richness–functional diversity association, SFD [32,41]). Further, different environmental drivers may selectively affect species according to their traits, either buffering or enhancing the slope of the SFD (i.e., increasing or decreasing extrinsic functional diversity [35]). Biotic interactions such as predation and facilitation, and abiotic conditions such as nutrient or water availability and temperature, can act as environmental filters. That is, they can select a relatively narrow set of traits from those available in the species pool, which results in consistently lower functional diversity than that expected from the SFD [32,35,42]. On the other hand, species interactions (such as competition) may promote trait differentiation (such as niche segregation, functional complementarity) and higher-than-expected functional diversity [43,44]. Thus, factors affecting the strength of those drivers may have consistently negative or positive effects on functional diversity, by either affecting species richness or directly constraining species composition according to their traits.

Therefore, assessing the relative importance of different environmental drivers on the functional diversity of species assemblages needs to distinguish the effects of factors affecting species richness from those affecting the size and magnitude of deviations of observed functional diversity from that predicted by the SFD [35,43,44]. Furthermore, temporal changes in functional diversity result from local extinction (or emigration) and colonization of species with different functional traits [14,35,44]. Such changes add traits to, or remove traits from, the existing assemblage. However, factors affecting functional diversity have seldom been analyzed in dynamic terms by autocorrelation in long-term time series [42,43]. For example, several studies have tested for regulation (i.e., negative autocorrelation) of species richness and ascribed their results to either niche processes or species pool limitations [45,46]. However, these studies largely ignored functional diversity, which is based on the traits that define species niches and has a well-defined association with the size and composition of the species pool [32,35,42,43,47].

Knowledge of the relative importance of different driving factors on functional diversity is greatest for plants, improving for animals, but still scarce for mammals [29,31–35,42]. Most publications rely on short-term, bibliographical data concerning functional traits and involve communities in the temperate region (two studies by Farias and Jaksic [42,43] are noteworthy exceptions). Research on animals to date has to a large extent neglected the tropics, the globe’s richest and most diverse region. Specifically, tropical dry forests are among the most threatened terrestrial ecosystems on Earth, subject to land-use changes, overexploitation, and extreme climate events resulting from climate change [37–39,48–53]. Understanding how these factors drive the diversity of tropical mammals using long-term biological and climate monitoring is a pivotal task we have not yet undertaken.

Under the strong pressure that current global climate disruption exerts on biodiversity (mainly involving habitat loss and fragmentation, species introduction and biotic homogenization), the most endangered species are those that live in ecosystems under threat, have restricted geographic distribution, and dispersal limitations (i.e., they are unable to travel through altitudinal or latitudinal gradients) [12,23–25,54–58]. Many tropical mammals are among these species, as most are small (which limits their dispersal), endemic to areas as small as few thousand square kilometers, and currently undergoing unprecedented habitat loss [1,37,38,59]. Yet some of these endemic species can exhibit unique functional traits. Thus, loosing endemic species not only erodes global diversity, but also can irreversibly impair functional diversity in tropical ecosystems.

Mexico is an ideal proving ground to understand how climate affects such diversity in a region rich in endemic mammals, thanks to geographical, historical, climatic and biotic factors over millennia [60–62]. More than 200 of its over 520 mammal species are endemic [39,60,63–65], with most being small mammals and over 70% rodents. These endemic animals include the only continental terrestrial carnivore endemic to Mexico, *Spilogale pygmaea* (pygmy spotted skunk), and one of the most restricted genuses of all mammals, *Xenomys nelsoni*, consisting of only a single species, Magdalena wood rat, both of which have a conservation status of “threatened” [60,61,66]. Mexico’s “hot spots” for endemic mammals are the southeastern rainforests and the Pacific slope dry tropical forests [60]. Unfortunately, these regions are suffering from extensive land use change, urbanization, and deforestation, causing the natural habitats for these species to decrease rapidly over the past 50–100 years [60–62,66]. Yet, most of our knowledge about the drivers of endemic mammal biodiversity in these hot spots are from short-term field data or studies.

In this paper, we endeavor to unravel the relative influence of different environmental drivers on the functional diversity of the small-mammal fauna of seasonal tropical dry forests of southwestern Mexico, a fauna rich in endemic species, using a dynamic approach to analyze long-term (19-year) time series. Particularly, we focused on the effects of seasonal and inter-annual variation in precipitation that characterize this ecosystem. Further, we took advantage of heterogeneity in vegetation cover and exposure to desiccation between two forest types (upland and arroyo or riparian forests), which are expected to differ in the strength of environmental filters.

We addressed the following five specific questions: **I**. Are the endemic species of small mammals from Western Mexico functionally redundant or, on the contrary, do they show distinct (complementary) ecological roles? **II**. Does functional diversity vary with species richness as expected from the SFD (i.e., random sorting from the species pool), or does it show patterns consistent with environmental filtering or interspecific competition? **III.** How do environmental factors affect deviation of observed functional diversity from SFD predictions? **IV**. Does the relative importance of these factors differ between habitats and/or seasons? **V**. Does functional diversity show evidence of regulation (negative autocorrelation)?

To assess the relative importance of biotic and abiotic factors as drivers of community structure, the observed functional diversity can be compared with that expected from random subsets of the species pool. Following Hansen *et al*. and Schindler [42,43], we inferred that competitive interaction (the limiting-similarity hypothesis) dominates the structure of small-mammal assemblages if observed values of functional diversity were consistently above those predicted by the null model (higher-than-expected functional diversity) for a given species richness level. In turn, we concluded that habitat or abiotic conditions (the environmental-filter hypothesis) constrain functional diversity if the observed values tended to consistently fall below those predicted by the null model (lower-than-expected functional diversity). If the observed functional diversity values were within those predicted by the null model, we concluded that they did not differ from chance, and no inference could be made about the mechanism behind the observed functional diversity.

## Methods and materials

### Setting

**Fig 1:**
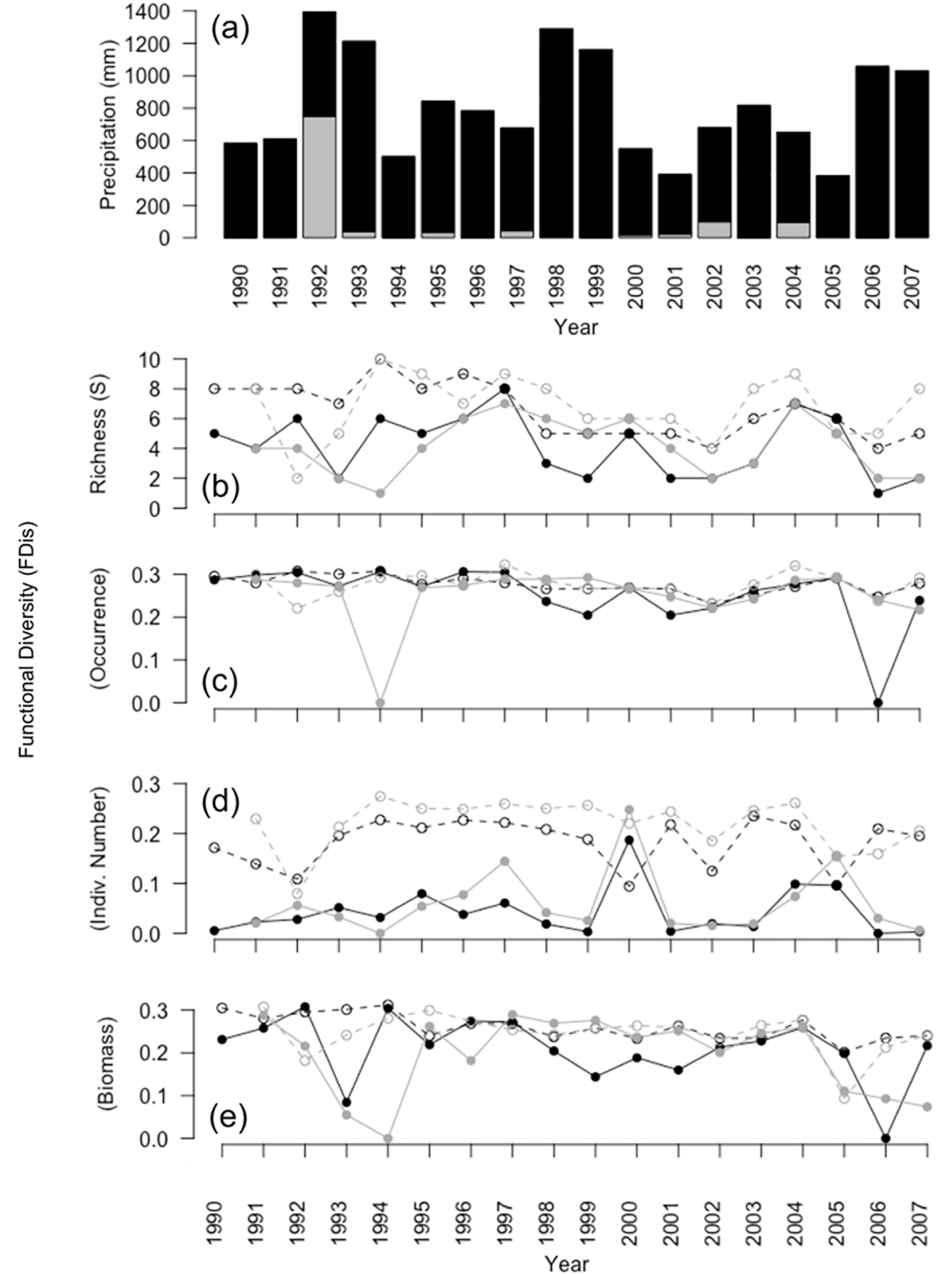
**Temporal dynamics of** (a) precipitation, (b) species richness, and functional diversity based on (c) species occurrence (incidence), (d) relative number of individuals, and (e) biomass, for the wet (black) and dry seasons (gray) for upland (continuous lines and filled dots) and arroyo forests (broken lines and open dots).

The climate of Chamela-Cuixmala Biosphere Reserve, a nature reserve on the Pacific coast in the state of Jalisco, Mexico (19°30′N, 105°03′W), is classified as a tropical wet-and-dry climate with summer showers and canicula [Awo (x′) I] in the Trewartha system, with 80% of rainfall on summer and fall (June to October), and a marked dry season, somewhat dry from November through February and severely dry from March to May [67,68]. The average annual rainfall from 1990 to 2007 plus 2012 was 850 ± 297mm (mean ± SD). The driest year of the time series was 2005, with rainfall of 383.8 mm, and the wettest was 1992 with over 1,393 mm (Fig 1A). In addition, there was a severe weather event in January 1992, resulting from atypical rainfall of 649 mm, which contrasts strongly with a January average of 35.3 ± 135.3 mm [67,68].

These seasons of extremely different precipitation cause dramatic phenological changes over the year. The Chamela-Cuixmala reserve includes over 13,000 ha of very well-preserved tropical dry forests; most of its area is occupied by upland forest, growing on the slopes, with young and shallow soils less than 1 m deep. Average tree canopy is around 10–15 m high, and tree species lose over 95% of their leaves during the dry season [68].

By contrast, the arroyo forests, the other prevalent vegetation type, are found in lowlands contiguous to upland forests, and their deeper (1–1.5 m), flat, alluvial and sandy soils retain more moisture than those of the upland forest. This allows tree canopies to reach 15–40 m high and to lose fewer leaves (50%–75% during the dry season) than trees in the upland forest [67].

Chamela-Cuixmala is a rare pristine tropical dry forest. Yet, the surrounding areas are subject to extensive deforestation, urbanization and pressure from cattle ranching. This context makes Chamela-Cuixmala an ideal study site for understanding the drivers of biodiversity in the absence of human-induced factors (others than global climate disruption). But, due to the extensive disturbance of the surrounding area, its biodiversity is highly vulnerable to local extinctions because of low potential for immigration or for rescue effects from similar habitats in the region.

### Field data methods

We monitored small mammals over 19 years (monthly from 1990 to1995 and bimonthly from 1996 to 2007 as well as in 2012) at six half-hectare sites, two sites in arroyo forest and four in the contiguous upland forest. All six sites were within the Chamela-Cuixmala Biological Station of the Universidad Nacional Autónoma de México, in Chamela-Cuixmala Biosphere Reserve. Mammals were captured using 64 Sherman traps baited with rolled oats mixed with peanut butter and vanilla). Traps were activated before dusk and were checked just after dawn to prevent heat stroke. This procedure also ensured that both nocturnal and diurnal species (shrews) could be captured and released without harm [69]. Animals captured in the traps were weighed using a metric Pesola^(R)^ conventional spring scale (from 10 g to 500 g, depending on the species captured), measured with a plastic metric ruler and released live on the same site where they were captured. All processes took no longer than five minutes per animal to avoid causing stress to the animal, and no animals were marked, sampled or killed. We strictly followed the American Society of Mammalogists guidelines for capturing, handling and releasing small wild mammals [69].

### Functional diversity analysis

To assess the functional diversity of the small mammals of the two contiguous tropical dry forests, a functional trait database was built from published information [70–72], and new data collected during the period of this study included data on the species’ habitat, stratum, diet, temporal activity, biomass and commonness (measured as average abundance). Field data provided information on mean biomass and abundance of small mammals, biomass is been proven related to functional diversity for other species [30]. Mean biomass was transformed to log10 to reduce the effect of the few relatively large species, and standardized by dividing by the standard deviation. Literature database was obtained by using IUCN [72] habitat categories, and defining species proportional use with information published by mammalogical compilations, just as the other three categories (habitat use (stratum), diet and temporal activity) [61,70,71]. Every variable was quantified into a proportion, for the sum of all their values, for every species, was 1. All data, on the functional traits used, available from the literature used was included.

**Fig 2:**
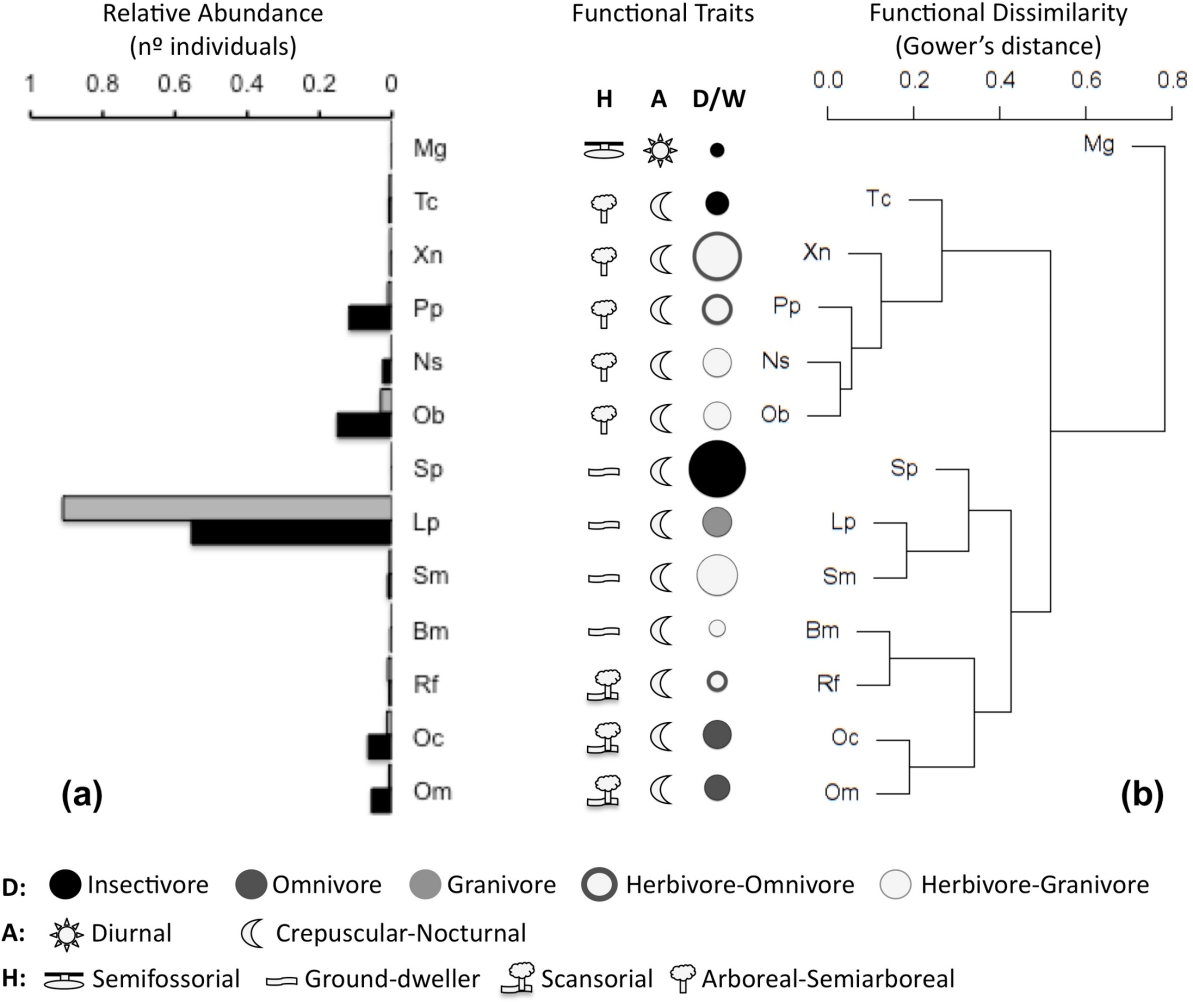
**Schematic summary of assemblage composition for small mammals in tropical dry forests of western Mexico**: (a) relative abundance for upland (black) and arroyo forests (gray), and (b) functional dissimilarity dendrogram and summary of functional traits for habitats: strata use (H), daily activity patterns (A), diet (D: circle color) and individual biomass (i.w.) weight, (W: proportional to circle size). Abbreviations for species: Bm (*Baiomys musculus*), Lp (*Liomys pictus)*, Mg (*Megasorex gigas)*, Ns (*Nyctomys sumichrasti)*, Oc (*Oryzomys mexicanus)*, Om (*Oryzomys melanotis*), Ob (*Osgoodomys banderanus)*, Pp (*Peromyscus perfulvus)*, Sm (*Sigmodon mascotensis)*, Sp (*Spilogale pygmaea*), Tc (*Tlacuatzin canescens)*, Xn (*Xenomys nelsoni)*. Further data in supplementary material (appendices).

Habitat use, habitat (stratum use), diet and temporal activity were quantified as multivariate traits, with a score assigned to different categories (e.g., nocturnal, crepuscular or diurnal for temporal activity; see details in S1 Table) according to their proportional use for each species. Because the relative use of the different categories of each multivariate trait are expected to show some degree of co-linearity, their dimensionality was reduced by performing a principal coordinates analysis (PCoA)), using the *capscale* function of the vegan R-package [73] in the R programming environment [74]. Previously, Euclidean distance matrices were obtained with the *dist* function of the vegan package [73]. Those axes explaining over 75% of the observed variability were retained as orthogonal functional traits, and included, along with mean biomass, as columns in a functional trait matrix (**T**). Then, a matrix of functional dissimilarity among species (**G**) was obtained using the Gower’s distance [30,42,75–78]:

gij=sum(Wk*Dijk)/sum(Wk)

where *Dijk* is dissimilarity (i.e., Euclidean distance) in (univariate or multivariate) functional trait *k* between species *j* and *i*, and *Wk* is a weighting factor inversely proportional to the total variance accounted for each (univariate or multivariate) functional trait *k*—*i*.*e*., *Wk* = 1 for the standardized mean biomass (univariate) and *Wk* = 1/*sk* for multivariate functional trait *k*, where *sk* is the total variance explained by the corresponding selected axes of the PCoA. Thus, each functional trait contributes similarly to functional differentiation among species (**G**) irrespective of its dimensionality (univariate or multivariate) and the original units used in their quantification. The resulting main pattern of functional dissimilarity among species is illustrated in the dendrogram (Fig 2B), which was obtained by applying an agglomerative-hierarchical cluster analysis on matrix **G**.

From matrix **G**, functional diversity was assessed yearly for each season (dry/wet) and forest type (upland/arroyo) using the functional dispersion (FDis) index [79], based on either (1) species occurrence (i.e., incidence as presence/absence, FDo), as an estimate of functional richness (i.e., the dispersion of functional traits present in the assemblage in the multivariate space), (2) the (relative) number of individuals of each species (FDn), or (3) the relative biomass of each species captured (FDw); the latter two included an evenness component [31]. FDn = FDo and FDw = FDo when functional evenness is highest (i.e., when species abundance is evenly distributed on the multivariate functional space), and FDn < FDo and/or FDw < FDo when functional evenness is lower. FDis estimates were obtained using the *dbFD* function in the FD R-package [80].

#### Determination of functional redundancy

To assess the level of functional redundancy in the assemblage, observed FDis values were contrasted against expectations from a null model that accounts only for species richness and the composition of the species pool (i.e., all species captured during the 19-year period). Null model predictions were calculated in R [74] by simulating 1,000 random extinction trajectories from the species pool. In each trajectory, species are subtracted from the pool one at a time, until only one is left (zero functional diversity), thus obtaining *S* − 1 random assemblages (where S represents species richness in the pool) [14,43]. Thus, 1,000 random assemblages were obtained for each level of species richness from 1 to *S* − 1, and the FDis indices were calculated for each of the assemblages, using the matrix G (see above, and S2–S9, Tables, S10 Table for acronyms). For each species richness level, the mean and distribution of the 1,000 FDis values represent the null-model prediction and the potential range for FDo (or FDn and FDw when evenness is maximal, see above).

The form of the random association between species richness and functional diversity (SFD) predicted by the model indicates the sensitivity of functional diversity to random species loss (i.e., the intrinsic functional redundancy [35]); a linear SFD indicates high functional complementarity (low intrinsic redundancy) and sensitivity to species loss, while an asymptotic association suggests high intrinsic redundancy and resilience to species loss.

Then, observed values of FDis were contrasted against null-model predictions to quantify deviations that could be attributed to environmental filters (i.e., higher-than expected redundancy) or species interactions (i.e., higher-than expected complementarity) [35,43,79]. In addition, FDis values predicted by the null model are expectations for FDo, and for FDn or FDw when evenness is maximal. Similar deviations for FDo (dFDo) and for FDn or FDw (dFDn or dFDw, respectively) indicate that dFDo values result mainly from species composition, whereas when dFDn < dFDo or dFDw < dFDo, uneven representation of species with different functional traits also contribute to differences between observed and expected functional diversity.

#### Modeling factors affecting functional diversity

Drivers of the temporal dynamics of functional diversity were assessed with auto-regressive linear models. Models were fitted separately for the dry and wet seasons of each year, and differences between forest types were assessed by including habitat (i.e., forest type [HAB]: upland vs. arroyo forest), and its interaction with environmental drivers, as additional factors in the model. As water availability constitutes a major limiting factor in dry forest ecosystems, the main tested environmental driver was seasonal precipitation of the current (PPt) and previous season (*PPt* − 1, i.e., previous dry season for wet-season data, and vice versa). In addition, seasonal averages of daily mean, maximum and minimum (T_MEAN_, T_MAX_, T_MIN_) temperatures were also included as predictor variables in the model. Finally, a dummy variable (YR92) was included to account for environmental effects associated with the anomalous precipitation recorded in the dry season of 1992, which not accounted for in the variables described above. Year was occasionally included to account for long-term temporal trends not accounted for by the environmental drivers.

Temporal variations in functional diversity may be caused by local colonization and extinctions of species (either true or apparent, due to changes in abundance and detection probability of rarer species) and subsequent changes in the repertory of functional traits present in the assemblage. These dynamics of additions and deletions of species from the assemblage may result in temporal autocorrelation with previous assemblage composition. Thus, the effects of environmental drivers may be best expressed as the net rate of change of biodiversity rather than on its resulting absolute value, and fitted models for any given biodiversity estimator Y take the following form:

Yt=α+Yt−1+B·Xt+βH·HABt+Ι·(HABt·Xt)+ε

or, rearranging:

ΔYt=Yt−Yt−1=α+B·Xt+βH·HABt+Ι·(HABt·Xt)+ε

where *Yt* and *Yt* − 1 are the value of the biodiversity parameter at the current and previous season, respectively, *ΔYt* is the resulting rate of change, α is a general intercept, ***X****t* is a matrix of N environmental drivers included as predictor variables for each time *t*, B and I are vectors of regression coefficients (i.e., B = {β_1_, β_2_, … β_N_}, I = {γ_1_, γ_2_, … γ_N_}) for environmental drivers and their interaction with habitat, respectively, and ε is a normal error term (ε = N(0,σ)).

Environmental drivers can directly affect functional diversity by constraining the presence or relative abundance of species with certain functional traits, or indirectly (through the SFD) by affecting species richness. Accordingly, to distinguish drivers affecting functional diversity directly or indirectly, we first modeled their effects on the net rate of change in species richness (ΔS), and then on the rate of change (ΔFDo, ΔFDn, and ΔFDw) of deviations from null model predictions of observed functional diversity based on species occurrence and on number and biomass of individuals (dFDo, dFDn, and dFDw, respectively). The latter models represent the influence of environmental drivers on functional diversity that it is not explained by the SFD, and that could enhance or buffer the effects of species loss or addition to the assemblage (i.e., decrease or increase its extrinsic functional redundancy [35]). Species richness (a counting variable) was normalized through log-transformation, and its rate of change took the following form:

ΔSt=log(St)−log(St−1)=log(St/St−1)

Species interactions may generate additional autocorrelation in the different biodiversity parameters if colonization, extinction or demographic responses depend on the occurrence and/or functional traits of other species. In particular, negative autocorrelation could indicate some degree of regulation in the dynamic of biodiversity, a pattern previously assessed for species richness but largely unexplored for functional diversity [42]. Thus, in modeling species richness and functional diversity dynamics, we included additional terms for species richness and both species richness and functional diversity (deviations from null-model predictions), respectively, in the previous season (S_t-1_, dFDo_t-1_, dFDn_t-1_, dFDw_t-1_) and the same season of the previous year (S_t-2_, dFDo_t-2_, dFDn_t-2_, dFDw_t-2_), along with their interaction with habitat.

Finally, population size and, thus, the number of individuals captured for each species vary greatly among years (Fig 1). This may affect the species richness and functional diversity due to under-representation of the less abundant species in samples. Thus, the total number of individuals captured for all species was included, log-transformed, as a co-variable in the models to account for spurious effects due to sample size.

Model selection was performed using the Akaike's information criterion corrected for small sample sizes (AICc) [81,82], and proceeded first using a backwards algorithm, starting with a full model and then deleting one variable at a time (first the interaction terms, and then the main factors) until a simple model was selected. Then, a stepwise procedure was used to improve the selected model, by inspecting the distribution and behavior of residuals, transforming variables when necessary, and testing whether adding variables decreased the AICc value. This procedure was repeated for all of the biodiversity parameters (species richness, and functional diversity based on occurrence, number of individuals and biomass) and the two seasons (dry/wet), resulting in eight selected models. To assess the relative importance of each predictor in the selected models, their partial determination coefficients were assessed as: *R*2*X* = *R*2 − *R*2 − *X*, where *R*2*X* is the partial determination coefficient for predictor *X*, *R*2 is the model’s determination coefficient, and *R*2 − *X* is the determination coefficient for the model without variable *X* its interactions.

## Results

### Species

We captured 13 species of small mammals during the 19 years of fieldwork, representing 66% of the mammalian species previously identified in the region [61,83]. The 13 species are: *Baiomys musculus* (southern pygmy mouse), *Liomys pictus (*painted spiny pocket mouse), *Megasorex gigas* (Mexican shrew), *Nyctomys sumichrasti*, (vesper rat), *Oryzomys melanotis* (black-eared rice rat), *Oryzomys mexicanus* (Coues’s rice rat), *Osgoodomys banderanus* (Michoacán deer mouse), *Peromyscus perfulvus* (tawny deer mouse), *Reithrodontomys fulvescens* (fulvous harvest mouse), *Sigmodon mascotensis* (west Mexican cotton rat), *Spilogale pygmaea* (pygmy spotted skunk), *Tlacuatzin canescens* (grayish mouse opossum) *and Xenomys nelsoni* (Magdalena wood rat). Fifty per cent of these species are endemic to Mexico, including three that are the only species in their genus (S1 Table). Overall, *L*. *pictus* was the numerically dominant species in the assemblage, reaching 90% of captures in upland forest, and 55% in arroyo forest, where abundance was more evenly distributed between this and other medium-sized (i.e., 35–40 g) species (Figs 1B and 2A).

#### Functional traits and demographic results

We created a database with the functional traits of every species (S1 Table, data on S11–S13 Tables), and obtained the arithmetical average weight for every species and the abundance for every species in both dry and wet seasons for the two habitats (S14 Table). Although both habitats share all the species (Fig 1B) and there was no difference in weight between habitats, there were striking differences in abundance between the two community structures (S12 and S13 Tables). In upland forest, over 90% of the individuals captured were *L*. *pictus*, while in arroyo forest, assemblages were more balanced, with only around 55% of the captures being *L*. *pictus* (Fig 2A).

#### Composition

The assemblage was numerically dominated by the medium-sized ground-dweller granivore *L*. *pictus*, with lower representation of omnivore scansorial species of the genus *Oryzomys* and of three medium-sized arboreal (unspecialized) herbivores (i.e., *P*. *perfulvus*, *O*. *banderanus* and *N*. *sumichrasti*) (Fig 2B). Extreme functional traits were poorly represented; largest, smallest and insectivore species were rare, with low functional diversity based on either number of individuals or biomass (i.e., evenness; Fig 2B). Among these rare species were both the largest (skunk *S*. *pygmaea*) and the smallest (shrew *M*. *gigas)* species, of which the latter is also the only diurnal and semifossorial small mammal in the assemblage (Fig 2B).

#### SFD and intrinsic functional redundancy

The dendrogram of functional dissimilarity indicates that most species show high redundancy (i.e., *gij* < 0.2) with one or more other species in the assemblage (Fig 2B); for example, the functional dissimilarity among the semiarboreal-arboreal and mainly herbivorous *O*. *banderanus*, *N*. *sumichrasti* and *P*. *perfulvus* was close to zero. Accordingly, although the null model for the SFD show a positive association, this was highly attenuated (i.e., asymptotic; S2 to S9 Figs), suggesting high levels of intrinsic functional redundancy for the entire small-mammal assemblage. Thus, random changes in species richness should have minor effects on functional diversity for assemblages with over five species.

### Environmental filtering

**Fig 3:**
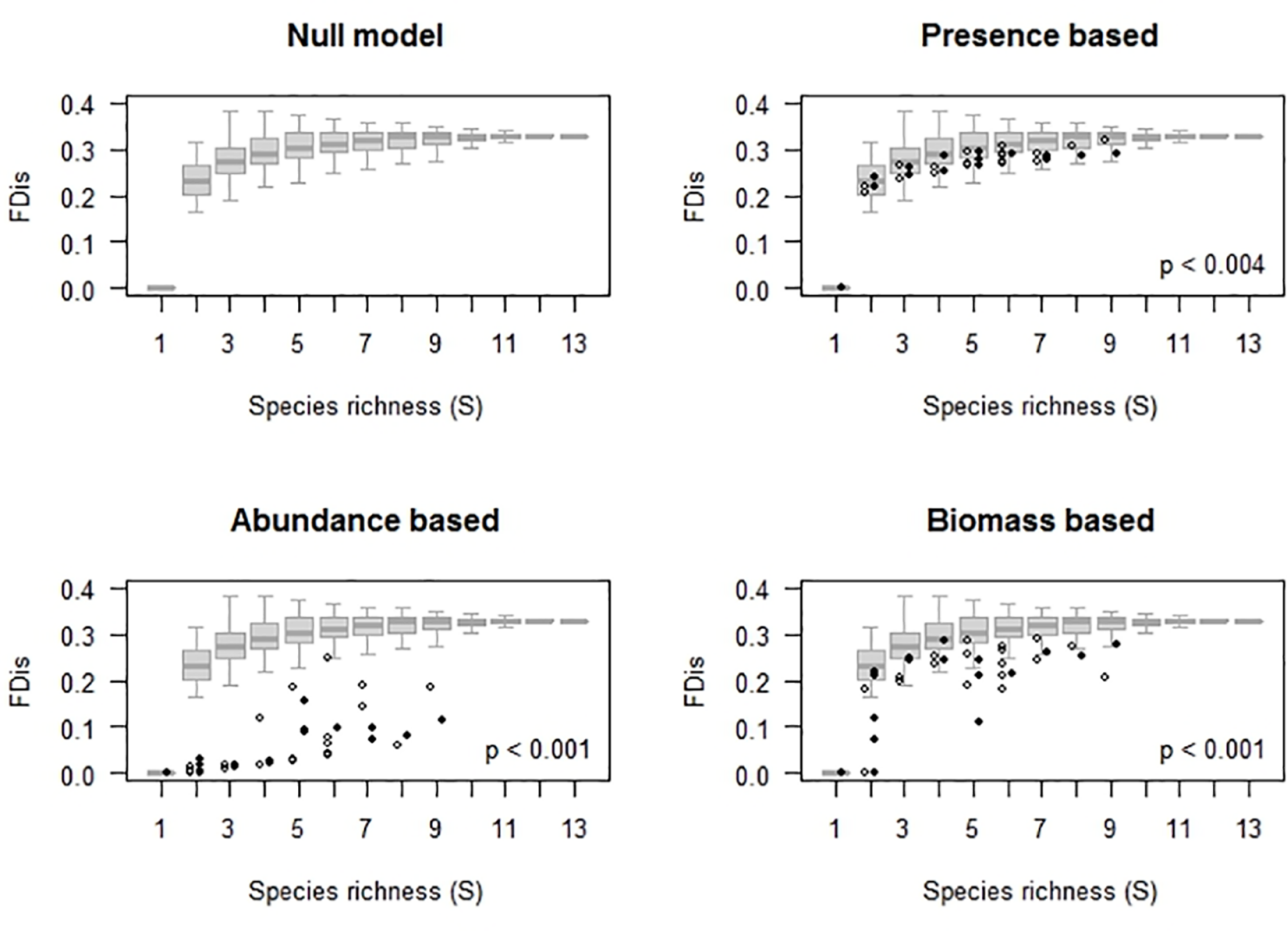
**Predictions for richness–functional diversity for upland forest** of the species richness–functional diversity association predicted by the null model (SFD) vs. observed functional diversity based on (b) species occurrence (incidence), (c) relative number of individuals, and (d) biomass. Field data for dry (open dots) and wet (filled dots) seasons.

**Fig 4:**
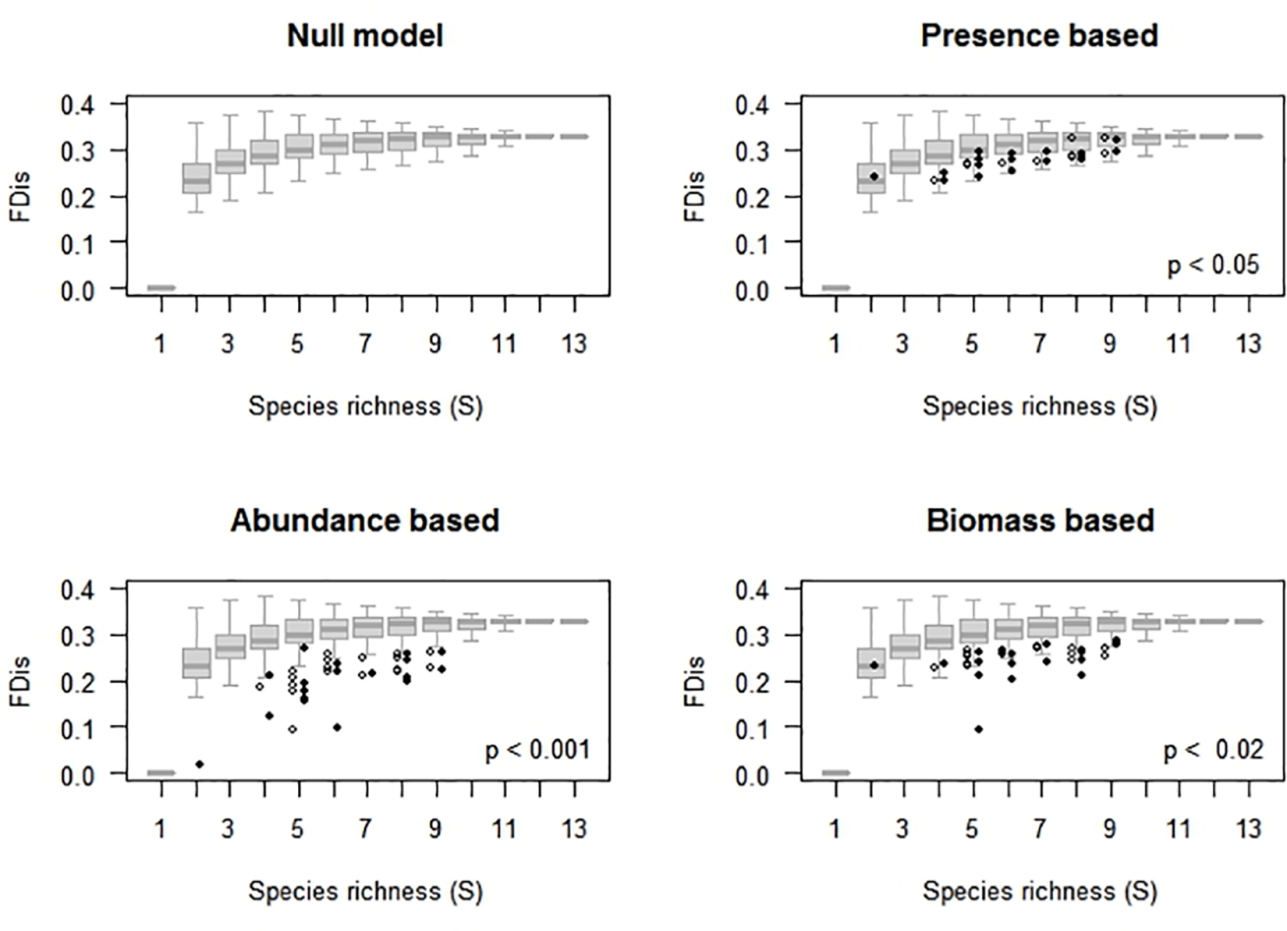
**Predictions for richness–functional diversity for arroyo forest species** of the species richness–functional diversity association predicted by the null model (SFD) vs. observed functional diversity based on (b) species occurrence (incidence), (c) relative number of individuals, and (d) biomass. Field data for dry (open dots) and wet (filled dots) seasons.

The three estimators of functional diversity (FDo, FDn and FDw) show negative deviations from the null model predictions in almost all cases (Figs 3 and 4), suggesting the effects of environmental filters. However, the magnitude of dFDo (functional richness, Figs 1B, 3 and 4, S3 and S4 Figs) was smaller than deviations of functional diversity based on species abundance (dFDn or dFDw, Figs 3 and 4, S5 and S6 Figs). This suggests low functional evenness: a non-uniform distribution of abundance in the multivariate functional space. In addition, dFDn was more negative than dFDw (Figs 3 and 4), particularly in the upland forest (Figs 1D and 3 and S5 Fig), indicating a relatively less uneven distribution for biomass than for the number of individuals. Such low functional evenness probably resulted from one (or several redundant) small-sized, numerically dominant species (Fig 2A and S8 and S9 Tables).

### Intrinsic dynamic of biodiversity

Arroyo forest shows less variability in all functional diversity indices (coefficient of variation [CV] = 23.45) than upland forest (CV = 64.27, Fig 1B–1E). In the upland forest, functional diversity based on species occurrence, number of individuals and biomass all crashed due to two extreme climate events in 1993–1994 and 2005–2006, whereas, for arroyo forest, functional diversity based on species occurrence and biomass exhibited only a mild decrease during these climate events (Fig 1C and 1E).

**Fig 5:**
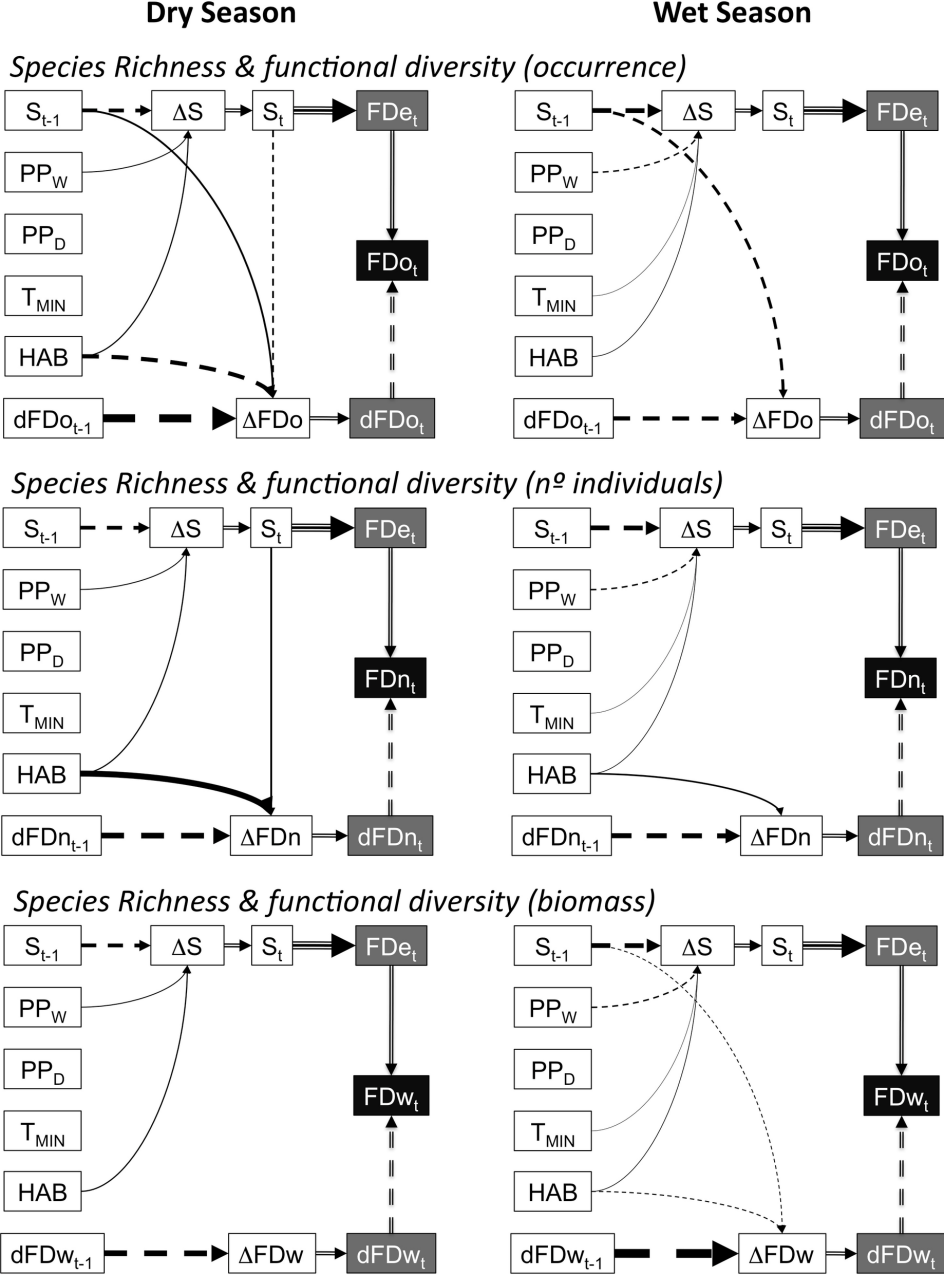
Summary of effects on each biodiversity component according to the selected models. For clarity, the effects of some fitting co-variables were omitted (e.g., temporal trends, sample-size effects), and only effects of interest are consistently shown. Positive (continuous lines) and negative (broken lines) effects are shown. Double-lined arrows are mathematical (not estimated) associations, and the triple-lined arrow between species richness (S_t_) and functional diversity predicted by the SFD (FDe_t_) represents the theoretical association between these variables predicted by the null model. Thus, the observed value for each biodiversity component (black box) is the sum of the effect of species richness predicted by the null model (i.e., intrinsic functional redundancy) and deviations from it (i.e., extrinsic functional redundancy), both represented by the gray boxes. Arrow width is proportional to the strength of the effects, according to the respective partial coefficient of determination. Acronyms and descriptions of variables are detailed in S10 Table.

Dynamic models show strong negative effects of values of the previous season on the rate of change of all biodiversity parameters (indicating first-order negative feedback on species richness and functional diversity based on species occurrence, number of individuals and biomass (Figs 3, 4 and 5 and S1–S9 Figs and S2–S9 Tables). This feedback explained about a third of the observed variance in the rate of change of species richness, and similar or even higher proportion (up to 65%) of variance in the rate of change for estimators of functional diversity (S3–S9 Tables). These effects suggest strong levels of regulation in the dynamics of small-mammal biodiversity, resulting either from competitive interactions among species or from a limited species (and traits) pool.

In addition, there were effects of species richness on functional diversity that differ depending on whether occurrence, number of individuals, abundance, or season is analyzed (Fig 5, S8 and S9 Tables). Species richness during the previous year negatively affected functional diversity based on occurrence and biomass in the wet season, and positively affected functional diversity based on occurrence in the dry season, while species richness during the current season had a negative effect on functional diversity based on occurrence and a positive effect on that based on number of individuals, both in the dry season.

### Differences between forest types

Forest type had variable effects on the dynamics of biodiversity parameters (Fig 5). Positive effects (i.e., higher values for upland forest) were found for species richness and functional diversity based on the number of individuals, while a marked negative effect (i.e., lower values for upland forest) was observed for functional diversity based on occurrence during the dry season, and a very slight negative effect for functional diversity based on biomass during the wet season. Overall, effects of forest type tended to be stronger during the dry season, suggesting higher differences between forest types during the more stressful season (Fig 5, S2 and S3 Tables). During this season, the inverse response of functional diversity based on occurrence and number of individuals indicates that species richness increases in upland forest due to the presence of relatively rare (less abundant) and functionally redundant species. This was particularly related to a higher representation of medium-sized (i.e., 35–40 g) scansorial-omnivore and arboreal-herbivore species, and a lower representation of the dominant medium-sized ground-dweller granivore *L*. *pictus* (Fig 2A, S14 Table).

### Other exogenous drivers of biodiversity

In addition to the endogenous dynamics (internal feedbacks) and forest-type effects, species richness was also affected by certain environmental factors (Fig 5, S2–S9 Tables). A higher minimum temperature favored species richness during the wet season, probably by reducing cold stress. In turn, wet-season precipitation had a positive effect on species richness in the dry season, likely a result of increased productivity during the preceding reproductive period, which positively affected the abundance and detection probability of rarer species, and a negative effect on species richness in the wet season, which could result from increased stress in the wettest years. Further, adding the exceptional rainy conditions during the dry season of 1992 as a dummy predictor variable improved model performance for the dry season by accounting for the low species richness value recorded in the upland forest in that year (Fig 1 and S3 and S8 Figs and S3 Table). Given that precipitation during the dry season did not result in a similar improvement in model performance, other environmental conditions affected by that unusual rainy event likely explain such a marked drop in species richness. However, none of these factors had systematic effects on functional diversity (Fig 5 and S2 and S3 Tables). This indicates that no recorded environmental variable selected species according to their functional traits, and thus no individual variable affects the strength of environmental filters.

### Accounting for potential sample-size bias

The effects of environmental variables on species richness and functional diversity can result from changes in colonization and extinction rates, or can be a consequence of changes in small-mammal abundance and in the associated probability of detection of rare species. However, the effect of sample size (i.e., number of individuals captured) is expected to vary with overall small-mammal abundance and, thus, productivity. This was explicitly accounted for in the modeling process, and significant effects of sample size were detected in some cases. The observed positive effect on species richness during the dry season, and the negative effects on dFDn in both seasons, suggest an increase in the probability of detection of very rare and functionally differentiated species as productivity increases, decreasing the unevenness in abundance distribution through the multivariate functional space. Further, the contrary effect on FDw during the wet season may result from an increase in the detection of larger functionally differentiated rare species, which led to a more even distribution of biomass over the multivariate space. Thus, after accounting for these effects of sample size, the detected effects of other variables were unlikely to be spurious associations due to abundance-related changes in detection of rare functionally differentiated species.

## Discussion

Western Mexico’s tropical dry forests are extraordinarily rich in endemic species, due to their geographical complexity, geological history, and climatic patterns [60–62,65]. Yet the effects of these climatic patterns have been poorly understood to date. Long-term monitoring of climate, and of the abundance and functional traits of small mammals, are needed to add to this knowledge, which might allow us to explore how climate—and its global disruption—affect ecosystem functioning. The present study addresses for the first time the extent of environmental forcing on taxonomic and functional diversity of an endangered small-mammal assemblage using a dynamic-modeling approach.

Ten of the 13 species (and three genera) modeled in this study are endemic to Mexico. This small-mammal assemblage rich in endemic species at Chamela-Cuixmala shows high levels of intrinsic functional redundancy, which is expected to confer some degree of resilience against random species loss [23,84,85]. However, the assemblage composition does not appear to respond randomly to changes in species number. In turn, observed functional diversity was consistently lower than expected by chance for any species richness level, irrespective of season and forest type, suggesting a dominant role of environmental filters [31,35,43]. In this context, water availability is a prevailing limiting factor for small-mammal populations in these dry ecosystems, particularly in the more exposed upland forest, where filtering effects were strongest [86].

Negative effects of environmental filters were more marked for estimates of functional diversity that accounted for the relative abundance and, thus, evenness of species, showing a high representation of few species with redundant functional traits, and rarity of those that are functionally distinct. In fact, the heteromyid *L*. *pictus* dominated the assemblage, thanks to physiological and ecological traits that allow the animal to cope with arid conditions, including cheek pouches, territorial behavior by males, strictly seed-feeding diet, and ability to concentrate urine [61,87]. These characteristics allow this species to be dominant in the upland forest, where dry seasons are harsher, constituting over 93% of the captures. The dominance of this single species reduced taxonomic and functional evenness in our sample.

In turn, dominance by *L*. *pictus* was less marked at the more mesic arroyo forest, where microclimatic conditions are less stressful [67]. Further, functional diversity in this habitat was less variable than in upland forest. Accordingly, the relatively small areas covered by arroyo forests may act as reservoirs of mammal species and functional diversity, allowing the recolonization of the most exposed and seasonal upland forest by rare and functionally differentiated species, particularly after extreme climate events.

Drivers of functional diversity could include stochastic processes [88,89], interspecies interactions [43,90] and environmental filtering [33,91]. According to our results, due to the strong seasonality exhibited by tropical dry forests, the community structure and functional diversity of the small mammals harbored within these forests rely on strong climatic filtering (rainfall oscillations). Although the main drivers were roughly the same for both habitats (upland and arroyo forests), their relative strength resulted in a gradient: the harsher the conditions, the stronger the effect of environmental filtering. Accordingly, environmental filters in upland forest have strong effects, resulting in one species (*L*. *pictus*) becoming dominant.

Environmental drivers, particularly precipitation, affected species richness, and, through it, the functional richness and diversity of the small-mammal assemblage. In other habitats, both tropical and temperate, research has found that temperature was a major driver of biodiversity fluctuations [21,22,92], but mean minimum temperature in our study site had only a minor effect on the species richness of the small-mammal assemblage. In this study, we found that the rainfall during the current wet season may have immediate negative effects on species richness, potentially through an increase in stress-induced mortality, but a positive effect in the following dry season, likely through increased productivity during reproduction. This kind of effect of rainfall has also been found for other small-mammal assemblages in arid lands in Chile [42,43,91] and other ecosystems [87,91,93]. In addition, effects of species richness on functional-diversity deviations from SFD predictions (dFDo, dFDn and dFDw) suggest that species interactions (e.g., interspecific competition) may play a role, but their actual relevance cannot be assessed from our analyses because of lack of consistency in the response and in the data to support it. Extreme climate events have been shown to change species patterns in desert rodents in Chihuahua, Mexico, favoring the extinction of dominant species and allowing opportunistic species to invade [13–15,87,94], although the effects on functional diversity are not known.

This understanding of the processes behind the functional richness and diversity of small mammals in Mexico’s tropical dry forests, together with the historical context of the region, helps explain the regional species diversity, both in Mexico and the tropics in general [1,36–38,66,95,96]. However, this region is subject to rapid change in land use and high levels of environmental pollution, which are leading to loss of natural vegetation at strikingly high rates, compromising the extent and structure of ecosystem services provided by these species [28,60,66,97]. The absence of a healthy small-mammal assemblage would have severe environmental consequences, as these animals may play fundamental roles in ecosystem processes in these tropical dry forests [53,63,97–99]. These findings stress the importance of increasing protected areas in these ecosystems, as they can buffer the effects of climate on natural populations. In this regard, thanks to its more moderate conditions, arroyo forest may be particularly important by providing habitat for small mammals evicted from the more extensive upland forest. In this study, we have shown that rainfall variability is a relevant factor for the stability and function of small-mammal assemblages in tropical dry forests. If climatic patterns become unstable, as projected by climate change models [4,12,16,100,101], these mainly endemic species would be at risk of extinction [25,53,58,60], and such extinctions would affect the whole ecosystem, as their ecological roles would be irremediably lost [13–15,31,58,102].

## Supporting information

S1 Fig Residual plots for the selected models for species richness. Black lines and dots are for upland forest and gray lines and dots are for arroyo forest. For the dry season, filled dots are for the dry season of 1992, characterized by unusually high levels of precipitation, the effect of which was statistically removed. For the wet season, continuous lines and filled dots are for period 1990–1997, while broken lines and open dots are for the period 1998–2007.
(PDF)

S2 Fig Temporal dynamics of observed species richness (dots and lines) and 95% confidence interval predicted by the selected models (gray area). For the dry season, filled dots represent the dry season of 1992, characterized by unusually high levels of precipitation. For the wet season, filled dots are for the period 1990–1997, open dots are for the period 1998–2007.
(PDF)

S3 Fig Residual plots for the selected models for deviations of functional diversity from null model predictions according to species occurrence (dFDo). Black lines and dots represents data for upland forest, while gray lines and dots are those for arroyo forest.
(PDF)

S4 Fig Temporal dynamics for the observed dFDo (dots and lines) and 95% confidence interval predicted by the selected models (gray area). (PDF)

S5 Fig Residual plots for the selected models for deviations of functional diversity from null model predictions according to number of individuals (dFDn). Black lines and dots represents data for upland forest, while gray lines and dots are those for arroyo forest.
(PDF)

S6 Fig Temporal dynamics for the observed dFDn (dots and lines) and 95% confidence interval predicted by the selected models (gray area). (PDF)

S7 Fig Residual plots for the selected models for deviations of functional diversity from null model predictions according to biomass (dFDw). Black lines and dots represents data for upland forest, while gray lines and dots are those for arroyo forest.
(PDF)

S8 Fig Temporal dynamics for the observed dFDw (dots and lines) and 95% confidence interval predicted by the selected models (gray area). (PDF)

S9 Fig Plots for residuals of the selected models for the four variables, back-transformed to species richness. Lines represent the expected association with the same variable in the previous season, after accounting for the effects of other variables. Black lines and dots represents data for upland forest, while gray lines and dots are those for arroyo forest. For species richness in the dry season, filled dots represent the dry season of 1992, characterized by unusually high levels of precipitation. For species richness in the wet season, continuous lines and filled dots correspond to period 1990–1997, while broken lines and open dots correspond to the period 1998–2007.
(PDF)

S1 Table Thirteen species of small mammals identified during the 19-year study of tropical dry forests in Mexico. (PDF)

S2 Table Model selection for the dynamics of species richness in the dry season. Results for the 30 best-performing models (i.e., lowest AICc values) are shown; the selected model is highlighted in bold. R^2^: determination coefficient, ΔAICc: difference between model’s AICc and the lowest AICc value, k: number of parameters fitted, n: sample size (i.e., time series length); for acronyms of variables, see S10 Table.
(PDF)

S3 Table Model selection for the dynamics of species richness in the wet season. Results for the 30 best-performing models (i.e., lowest AICc values) are shown; the selected model is highlighted in bold. R^2^: determination coefficient, ΔAICc: difference between model’s AICc and the lowest AICc value, k: number of parameters fitted, n: sample size (i.e., time series length); for acronyms of variables, see S10 Table.
(PDF)

S4 Table Model selection for the dynamics of deviations of functional diversity (according to species’ occurrence) from null model expectations in the dry season. Results for the 30 best-performing models (i.e., lowest AICc values) are shown; the selected model is highlighted in bold type. R^2^: determination coefficient, ΔAICc: difference between model’s AICc and the lowest AICc value, k: number of parameters fitted, n: sample size (i.e., time series length); for acronyms of variables, see S10 Table.
(PDF)

S5 Table Model selection for the dynamics of deviations of functional diversity (according to species’ occurrence) from null model expectations in the wet season. Results for the 30 best-performing models (i.e., lowest AICc values) are shown; the selected model is highlighted in bold. R^2^: determination coefficient, ΔAICc: difference between model’s AICc and the lowest AICc value, k: number of parameters fitted, n: sample size (i.e., time series length); for acronyms of variables, see S10 Table.
(PDF)

S6 Table Model selection for the dynamics of deviations of functional diversity (according to number of individuals) from null model expectations in the dry season. Results for the 30 best-performing models (i.e., lowest AICc values) are shown; the selected model is highlighted in bold. R^2^: determination coefficient, ΔAICc: difference between model’s AICc and the lowest AICc value, k: number of parameters fitted, n: sample size (i.e., time series length); for acronyms of variables, see S10 Table.
(PDF)

S7 Table Model selection for the dynamics of deviations of functional diversity (according to number of individuals) from null model expectations in the wet season. Results for the 30 best-performing models (i.e., lowest AICc values) are shown; the selected model is highlighted in bold. R^2^: determination coefficient, ΔAICc: difference between model's AICc and the lowest AICc value, k: number of parameters fitted, n: sample size (i.e., time series length); for acronyms of variables, see S10 Table.
(PDF)

S8 Table Model selection for the dynamic of deviations of functional diversity (according to biomass) from null model expectations in the dry season. Results for the 30 best-performing models (i.e., lowest AICc values) are shown; the selected model is highlighted in bold. R^2^: determination coefficient, ΔAICc: difference between model’s AICc and the lowest AICc value, k: number of parameters fitted, n: sample size (i.e., time series length); for acronyms of variables, see S10 Table.
(PDF)

S9 Table Model selection for the dynamic of deviations of functional diversity (according to biomass) from null model expectations in the wet season. Results for the 30 best-performing models (i.e., lowest AICc values) are shown; the selected model is highlighted in bold. R^2^: determination coefficient, ΔAICc: difference between model’s AICc and the lowest AICc value, k: number of parameters fitted, n: sample size (i.e., time series length); for acronyms of variables, see S10 Table.
(PDF)

S10 Table Acronyms and short description of variables in S7 to S14 Tables. (PDF)

S11 Table Functional traits database. The traits on every category (habitat, habitat (stratum) use, diet and temporal activity) are multivariate variables standardized so the sum of the variables on every category sum 1. Habitat was obtained from IUCN (and its category numbers are included) [72], proportions of use were obtained from compilations, just as the variables in the other three categories [61,70,71,83]. Abbreviations are: Bm (*Baiomys musculus)*, *Lp (Liomys pictus)*, *Mg (Megasorex gigas)*, *Ns (Nyctomys sumichrasti)*, *Oc (Oryzomys mexicanus)*, *Om (Oryzomys melanotis)*, *Ob (Osgoodomys banderanus*, *Pp (Peromyscus perfulvus)*, *Rf (Reithrodontomys fulvescens)*, *Sm (Sigmodon mascotensis)*, *Sp (Spilogale pygmaea)*, *Tc (Tlacuatzin canescens)*, *Xn (Xenomys nelsoni)*.
(PDF)

S12 Table Abundance database upland forest. Abbreviations are: Bm (*Baiomys musculus)*, *Lp (Liomys pictus)*, *Mg (Megasorex gigas)*, *Ns (Nyctomys sumichrasti)*, *Oc (Oryzomys mexicanus)*, *Om (Oryzomys melanotis)*, *Ob (Osgoodomys banderanus*, *Pp (Peromyscus perfulvus)*, *Rf (Reithrodontomys fulvescens)*, *Sm (Sigmodon mascotensis)*, *Sp (Spilogale pygmaea)*, *Tc (Tlacuatzin canescens)*, *Xn (Xenomys nelsoni)*. *D (Dry season)*, *W (wet season)*.
(PDF)

S13 Table Abundance database arroyo forest. Abbreviations are: Bm (*Baiomys musculus)*, *Lp (Liomys pictus)*, *Mg (Megasorex gigas)*, *Ns (Nyctomys sumichrasti)*, *Oc (Oryzomys mexicanus)*, *Om (Oryzomys melanotis)*, *Ob (Osgoodomys banderanus*, *Pp (Peromyscus perfulvus)*, *Rf (Reithrodontomys fulvescens)*, *Sm (Sigmodon mascotensis)*, *Sp (Spilogale pygmaea)*, *Tc (Tlacuatzin canescens)*, *Xn (Xenomys nelsoni)*. *D (Dry season)*, *W (wet season)*.
(PDF)

S14 Table Average weight of species. Abbreviations are: Bm (*Baiomys musculus)*, *Lp (Liomys pictus)*, *Mg (Megasorex gigas)*, *Ns (Nyctomys sumichrasti)*, *Oc (Oryzomys mexicanus)*, *Om (Oryzomys melanotis)*, *Ob (Osgoodomys banderanus*, *Pp (Peromyscus perfulvus)*, *Rf (Reithrodontomys fulvescens)*, *Sm (Sigmodon mascotensis)*, *Sp (Spilogale pygmaea)*, *Tc (Tlacuatzin canescens)*, *Xn (Xenomys nelsoni)*.
(PDF)
