# Supplementary material for: Two decades of climate driving the dynamics of functional and taxonomic diversity of a tropical small mammal community in western Mexico
Source: PLoS One. 2017 Dec 11;12(12):e0189104. doi: 10.1371/journal.pone.0189104 (PMC5724848; doi:10.1371/journal.pone.0189104)

S1 Fig: Residual plots for the selected models for species richness. Black lines and dots are for upland forest and gray lines and dots are for arroyo forest. For the dry season, filled dots are for the dry season of 1992, characterized by unusually high levels of precipitation, the effect of which was statistically removed. For the wet season, continuous lines and filled dots are for period 1990–1997, while broken lines and open dots are for the period 1998–2007.

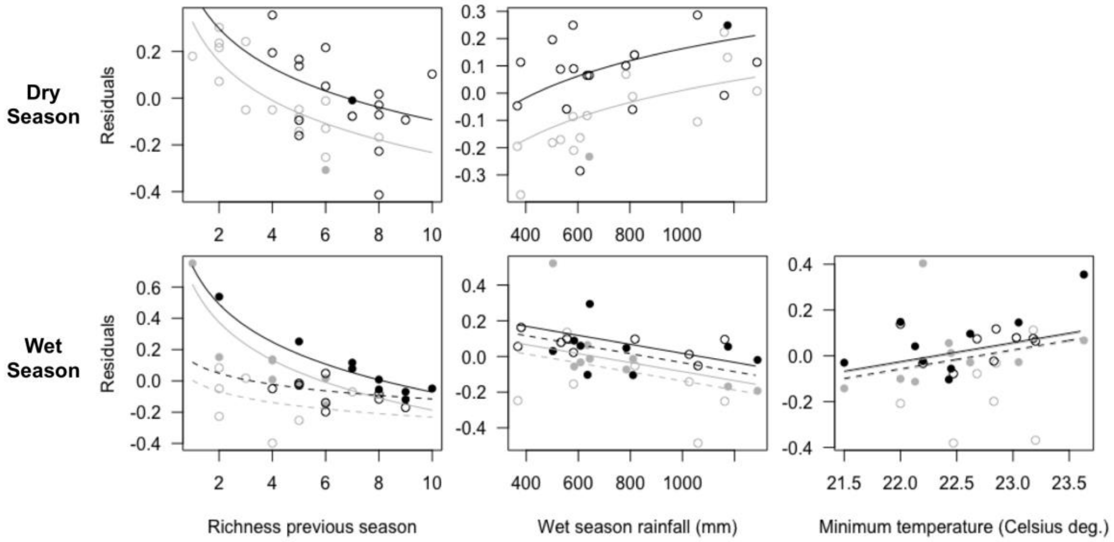

Supplement: S1 Fig — Black lines and dots are for upland forest and gray lines and dots are for arroyo forest. For the dry season, filled dots are for the dry season of 1992, characterized by unusually high levels of precipitation, the effect of which was statistically removed. For the wet season, continuous lines and filled dots are for period 1990–1997, while broken lines and open dots are for the period 1998–2007. (PDF) [file pone.0189104.s001.pdf]
