# Supplementary material for: Two decades of climate driving the dynamics of functional and taxonomic diversity of a tropical small mammal community in western Mexico
Source: PLoS One. 2017 Dec 11;12(12):e0189104. doi: 10.1371/journal.pone.0189104 (PMC5724848; doi:10.1371/journal.pone.0189104)

S2 Fig: Temporal dynamics of observed species richness (dots and lines) and 95% confidence interval predicted by the selected models (gray area). For the dry season, filled dots represent the dry season of 1992, characterized by unusually high levels of precipitation. For the wet season, filled dots are for the period 1990–1997, open dots are for the period 1998–2007.

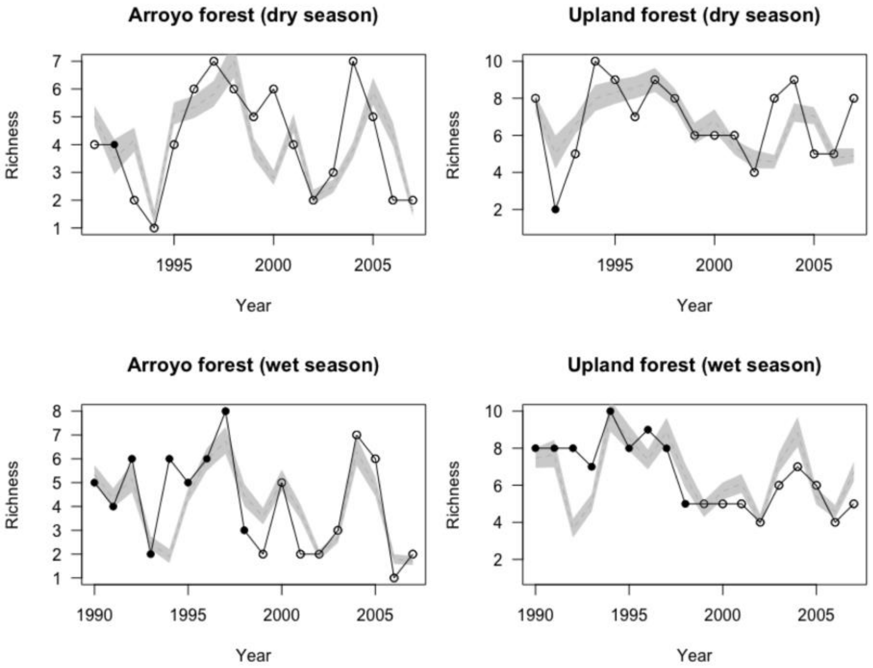

Supplement: S2 Fig — For the dry season, filled dots represent the dry season of 1992, characterized by unusually high levels of precipitation. For the wet season, filled dots are for the period 1990–1997, open dots are for the period 1998–2007. (PDF) [file pone.0189104.s002.pdf]
