# Supplementary material for: Two decades of climate driving the dynamics of functional and taxonomic diversity of a tropical small mammal community in western Mexico
Source: PLoS One. 2017 Dec 11;12(12):e0189104. doi: 10.1371/journal.pone.0189104 (PMC5724848; doi:10.1371/journal.pone.0189104)

S3 Fig: Residual plots for the selected models for deviations of functional diversity from null model predictions according to species occurrence (dFDo). Black lines and dots represents data for upland forest, while gray lines and dots are those for arroyo forest.

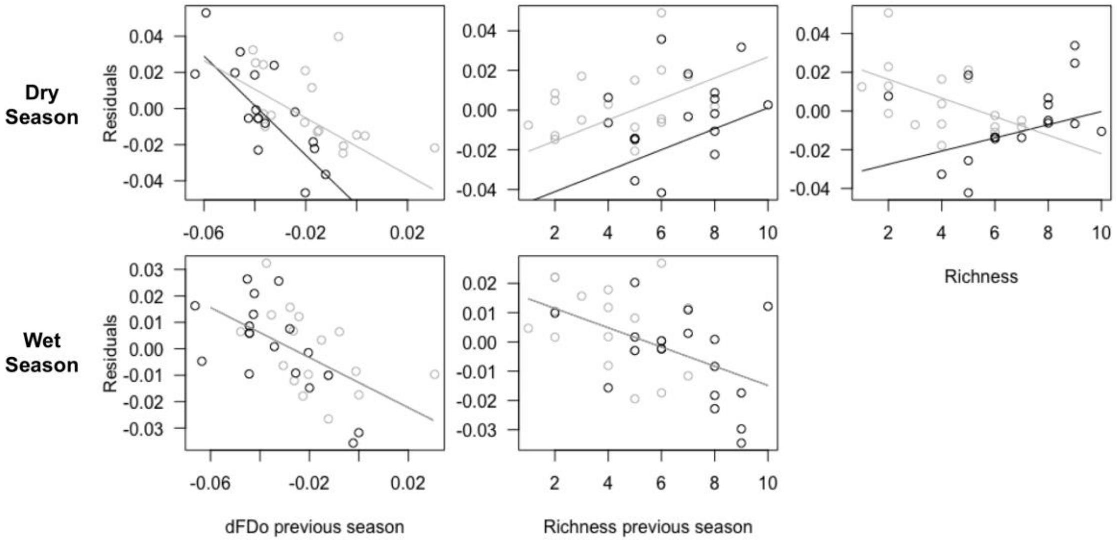

Supplement: S3 Fig — Black lines and dots represents data for upland forest, while gray lines and dots are those for arroyo forest. (PDF) [file pone.0189104.s003.pdf]
