# Supplementary material for: Two decades of climate driving the dynamics of functional and taxonomic diversity of a tropical small mammal community in western Mexico
Source: PLoS One. 2017 Dec 11;12(12):e0189104. doi: 10.1371/journal.pone.0189104 (PMC5724848; doi:10.1371/journal.pone.0189104)

**S5 Fig: Residual plots for the selected models for deviations of functional diversity** from null model predictions according to number of individuals (dFDn). Black lines and dots represents data for upland forest, while gray lines and dots are those for arroyo forest.

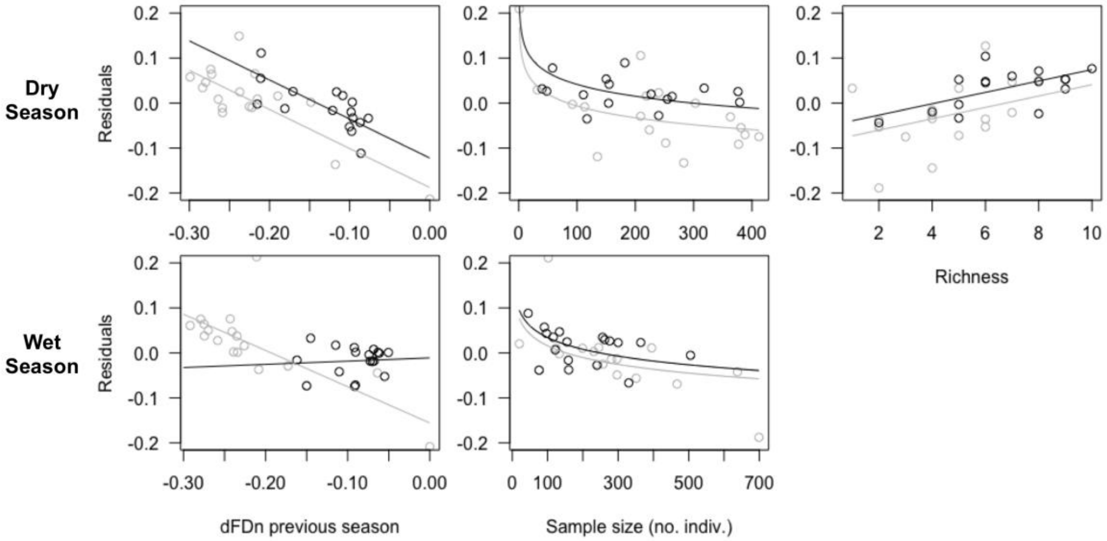

Supplement: S5 Fig — Black lines and dots represents data for upland forest, while gray lines and dots are those for arroyo forest. (PDF) [file pone.0189104.s005.pdf]
