# Supplementary material for: Two decades of climate driving the dynamics of functional and taxonomic diversity of a tropical small mammal community in western Mexico
Source: PLoS One. 2017 Dec 11;12(12):e0189104. doi: 10.1371/journal.pone.0189104 (PMC5724848; doi:10.1371/journal.pone.0189104)

S6 Fig: Temporal dynamics for the observed dFDn (dots and lines) and 95% confidence interval predicted by the selected models (gray area).

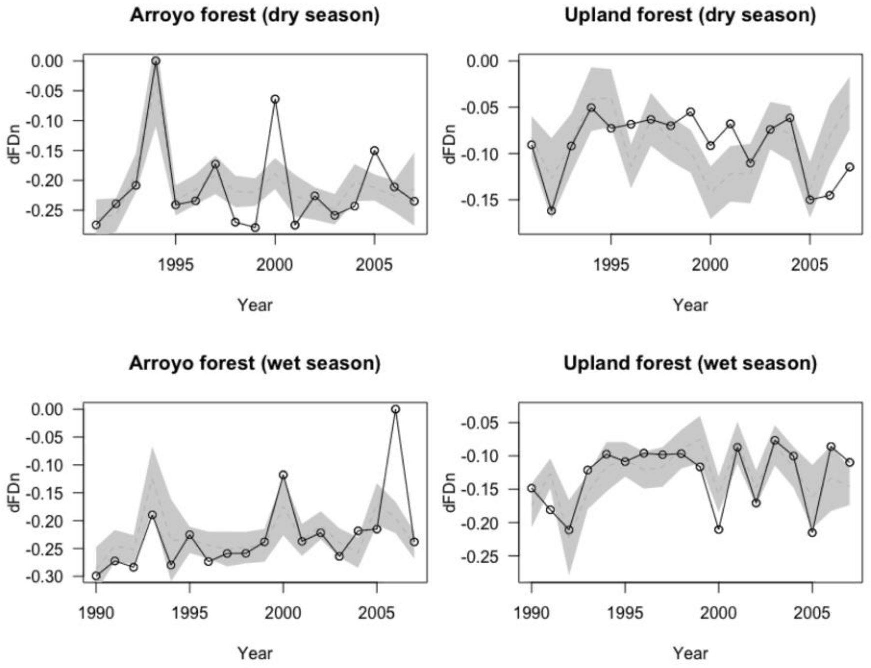

Supplement: S6 Fig — (PDF) [file pone.0189104.s006.pdf]
