# Supplementary material for: Two decades of climate driving the dynamics of functional and taxonomic diversity of a tropical small mammal community in western Mexico
Source: PLoS One. 2017 Dec 11;12(12):e0189104. doi: 10.1371/journal.pone.0189104 (PMC5724848; doi:10.1371/journal.pone.0189104)

S9 Fig: Plots for residuals of the selected models for the four variables, back-transformed to species richness. Lines represent the expected association with the same variable in the previous season, after accounting for the effects of other variables. Black lines and dots represents data for upland forest, while gray lines and dots are those for arroyo forest. For species richness in the dry season, filled dots represent the dry season of 1992, characterized by unusually high levels of precipitation. For species richness in the wet season, continuous lines and filled dots correspond to period 1990–1997, while broken lines and open dots correspond to the period 1998–2007.

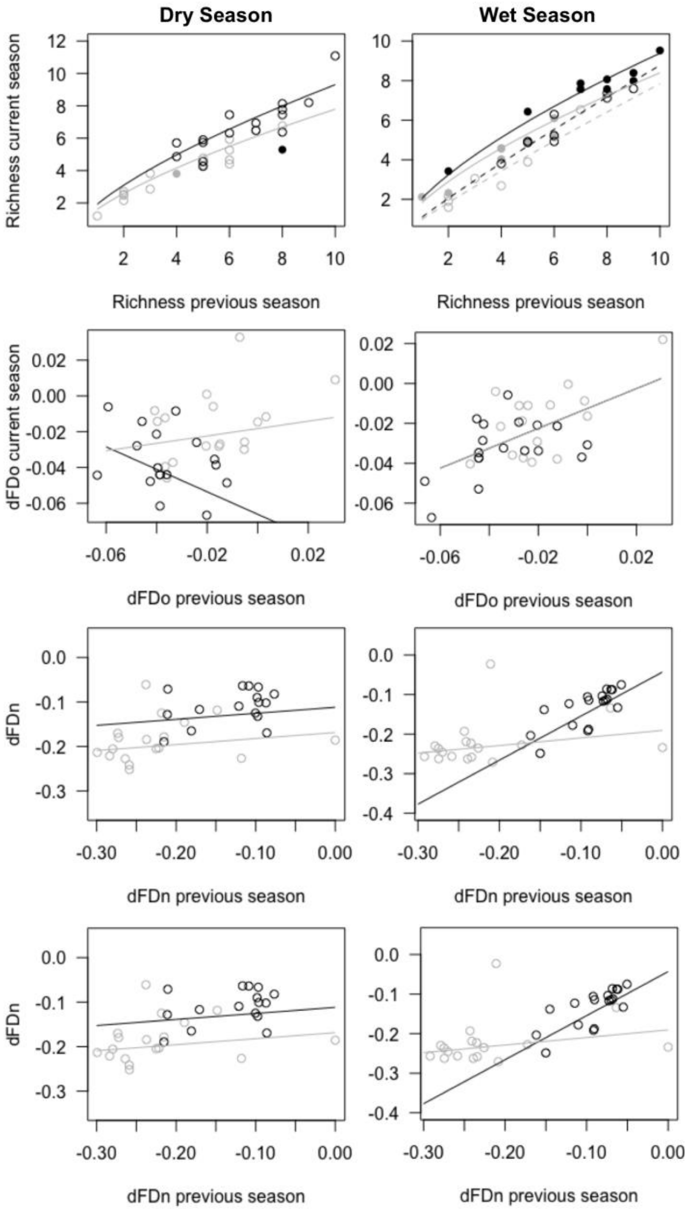

Supplement: S9 Fig — Lines represent the expected association with the same variable in the previous season, after accounting for the effects of other variables. Black lines and dots represents data for upland forest, while gray lines and dots are those for arroyo forest. For species richness in the dry season, filled dots represent the dry season of 1992, characterized by unusually high levels of precipitation. For species richness in the wet season, continuous lines and filled dots correspond to period 1990–1997, while broken lines and open dots correspond to the period 1998–2007. (PDF) [file pone.0189104.s009.pdf]
