# Supplementary material for: Two decades of climate driving the dynamics of functional and taxonomic diversity of a tropical small mammal community in western Mexico
Source: PLoS One. 2017 Dec 11;12(12):e0189104. doi: 10.1371/journal.pone.0189104 (PMC5724848; doi:10.1371/journal.pone.0189104)
Supplement: S1 Table — (PDF) [file pone.0189104.s010.pdf]

S1 Table : Thirteen species of small mammals identified during the 19-year study of tropical dry forests in Mexico

Order	Family	Species (scientific/English)	Endemicity	Conservation status
Didelphimorphia	Didelphidae	(Tc) *Tlacuatzin canescens*<br>Grayish Mouse Opossum[a]	Mexico[a]	Least concern
Soricomorpha	Soricidae	(Mg) *Megasorex gigas*<br>Mexican Shrew	Mexico[a]	Threatened
Carnivora	Mephitidae	(Sm) *Spilogale pygmaea*<br>Pygmy Spotted Skunk	Mexico	Threatened
Rodentia	Heteromyidae	(Lp) *Liomys pictus*<br>Painted Spiny Pocket Mice	Central America<br>and Mexico	Least concern
Rodentia	Muridae	(Bm) *Baiomys musculus*<br>Southern Pygmy mouse	Central America<br>and Mexico	Least concern
Rodentia	Muridae	(Ns) *Nyctomys sumichrasti*<br>Vesper Rat	Central America<br>and Mexico	Least concern
Rodentia	Muridae	(Oc) *Oryzomys mexicanus*<br>Coues' Rice Rat	Mexico	Not assessed
Rodentia	Muridae	(Om) *Oryzomys melanotis*<br>Black-eared Rice Rat	Mexico	Least concern
Rodentia	Muridae	(Ob) *Osgoodomys banderanus*<br>Michoacan Deer Mouse[a]	Mexico[a]	Least concern
Rodentia	Muridae	(Pp) *Peromyscus perfulvus*<br>Tawny Deer Mouse	Mexico	Least concern
Rodentia	Muridae	(Rf) *Reithrodontomys fulvescens*<br>Fulvous Harvest Mouse	North America	Least concern
Rodentia	Muridae	(Sm) *Sigmodon mascotensis*<br>West Mexican Cotton Rat	Mexico	Least concern
Rodentia	Muridae	(Xn) *Xenomys nelsoni*<br>Magdalena Wood Rat[a]	Mexico[a]	Threatened

[a] Species that are the sole species in their genus, which is also endemic to Mexico.
