# Supplementary material for: Two decades of climate driving the dynamics of functional and taxonomic diversity of a tropical small mammal community in western Mexico
Source: PLoS One. 2017 Dec 11;12(12):e0189104. doi: 10.1371/journal.pone.0189104 (PMC5724848; doi:10.1371/journal.pone.0189104)
Supplement: S2 Table — Results for the 30 best-performing models (i.e., lowest AICc values) are shown; the selected model is highlighted in bold. R2: determination coefficient, ΔAICc: difference between model’s AICc and the lowest AICc value, k: number of parameters fitted, n: sample size (i.e., time series length); for acronyms of variables, see S10 Table. (PDF) [file pone.0189104.s011.pdf]

S2 Table: Model selection for the dynamics of species richness in the dry season. Results for the 30 best-performing models (i.e., lowest AICc values) are shown; the selected model is highlighted in bold. R^2: determination coefficient, ΔAICc: difference between model's AICc and the lowest AICc value, k: number of parameters fitted, n: sample size (i.e., time series length); for acronyms of variables, see S10 Table.

Model	R^2	ΔAICc	k	n
**$\Delta S \sim \log(S_{t-1}) + YR_{92} + \log(PP_W) + HAB + \log(N)$**	**0.80**	**0**	**6**	**34**
$\Delta S \sim \log(S_{t-1}) + YR_{92} + PP_W + HAB + PP_W \times HAB + YR_{92} \times HAB + \log(N)$	0.83	0.2	8	34
$\Delta S \sim \log(S_{t-1}) + YR_{92} + PP_W + HAB + PP_W \times HAB + \log(N)$	0.81	0.7	7	34
$\Delta S \sim \log(S_{t-1}) + YR_{92} + PP_W + HAB + \log(N)$	0.79	1.3	6	34
$\Delta S \sim \log(S_{t-1}) + YR_{92} + \log(PP_W) + HAB + \log(N) + T_{MAX} + T_{MAX} \times HAB$	0.83	1.4	8	34
$\Delta S \sim \log(S_{t-1}) + PP_D + PP_W + HAB + PP_W \times HAB + \log(N)$	0.81	1.5	7	34
$\Delta S \sim \log(S_{t-1}) + YR_{92} + PP_W + HAB + YR_{92} \times HAB + \log(N)$	0.81	1.5	7	34
$\Delta S \sim \log(S_{t-1}) + PP_D + PP_W + HAB + \log(N)$	0.78	1.9	6	34
$\Delta S \sim \log(S_{t-1}) + YR_{92} + \log(PP_W) + HAB + \log(N) + T_{MAX}$	0.80	2.2	7	34
$\Delta S \sim \log(S_{t-1}) + YR_{92} + \log(PP_W) + \log(PP_W)^2 + HAB + \log(N)$	0.80	2.5	7	34
$\Delta S \sim \log(S_{t-1}) + YR_{92} + PP_W + PP_W^2 + HAB + \log(N)$	0.80	2.6	7	34
$\Delta S \sim \log(S_{t-1}) + YR_{92} + \log(PP_W) + HAB + \log(N) + T_{MEAN} + T_{MEAN} \times HAB$	0.82	2.6	8	34
$\Delta S \sim S_{t-1} + PP_D + PP_W + HAB + PP_W \times HAB + \log(N)$	0.80	2.7	7	34
$\Delta S \sim S_{t-1} + PP_D + PP_W + HAB + PP_D \times HAB + PP_W \times HAB + \log(N)$	0.82	2.8	8	34
$\Delta S \sim \log(S_{t-1}) + YR_{92} + \log(PP_W) + HAB + \log(N) + S_{t-2}$	0.80	3.0	7	34
$\Delta S \sim \log(S_{t-1}) + YR_{92} + \log(PP_W) + HAB + \log(N) + T_{MEAN}$	0.80	3.3	7	34
$\Delta S \sim \log(S_{t-1}) + YR_{92} + \log(PP_W) + HAB + \log(N) + T_{MIN}$	0.80	3.4	7	34
$\Delta S \sim \log(S_{t-1}) + PP_D + PP_W + PP_W^2 + HAB + PP_W \times HAB + \log(N)$	0.82	3.7	8	34
$\Delta S \sim \log(S_{t-1}) + PPD + \log(PP_W) + HAB + PPW \times HAB + \log(N)$	0.81	3.9	7	34
$\Delta S \sim \log(S_{t-1}) + PP_D + PP_D^2 + PP_W + HAB + PP_W \times HAB + \log(N)$	0.81	4.0	7	34
$\Delta S \sim \log(S_{t-1}) + PP_D + YR_{92} + PP_W + HAB + PP_W \times HAB + \log(N)$	0.81	4.0	8	34
$\Delta S \sim S_{t-1} + PP_D + \log(PP_W) + HAB + PP_W \times HAB + \log(N)$	0.81	4.1	7	34
$\Delta S \sim \log(S_{t-1}) + YR_{92} + \log(PP_W) + HAB + \log(N) + T_{MIN} + T_{MIN} \times HAB$	0.81	4.1	8	34
$\Delta S \sim S_{t-1} + PP_D + PP_W + HAB + S_{t-1} \times HAB + PP_W \times HAB + \log(N)$	0.81	5.2	8	34
$\Delta S \sim \log(S_{t-1}) + YR_{92} + \log(PP_W) + HAB + \log(N) + S_{t-2} + S_{t-2} \times HAB$	0.81	5.4	8	34
$\Delta S \sim S_{t-1} + PP_D + PP_W + HAB + S_{t-1} \times HAB + PP_D \times HAB + PP_W \times HAB + \log(N)$	0.83	5.5	9	34
$\Delta S \sim \log(S_{t-1}) + YR_{92} + \log(PP_W) + HAB + \log(N) + \log(S_{t-2}) + \log(S_{t-2}) \times HAB$	0.80	5.5	8	34
$\Delta S \sim \log(S_{t-1}) + YR_{92} + \log(PP_W) + HAB + \log(N) + \log(S_{t-2}) + \log(S_{t-2}) \times HAB$	0.80	5.5	8	34
$\Delta S \sim S_{t-1} + PP_D + PP_W + HAB + S_{t-1} \times HAB + \log(N)$	0.78	5.7	7	34
$\Delta S \sim S_{t-1} + PP_D + PP_W + HAB + \log(N)$	0.76	5.8	6	34
