# Supplementary material for: Two decades of climate driving the dynamics of functional and taxonomic diversity of a tropical small mammal community in western Mexico
Source: PLoS One. 2017 Dec 11;12(12):e0189104. doi: 10.1371/journal.pone.0189104 (PMC5724848; doi:10.1371/journal.pone.0189104)
Supplement: S3 Table — Results for the 30 best-performing models (i.e., lowest AICc values) are shown; the selected model is highlighted in bold. R2: determination coefficient, ΔAICc: difference between model’s AICc and the lowest AICc value, k: number of parameters fitted, n: sample size (i.e., time series length); for acronyms of variables, see S10 Table. (PDF) [file pone.0189104.s012.pdf]

S3 Table: Model selection for the dynamics of species richness in the wet season. Results for the 30 best-performing models (i.e., lowest AICc values) are shown; the selected model is highlighted in bold. R^2: determination coefficient, ΔAICc: difference between model's AICc and the lowest AICc value, k: number of parameters fitted, n: sample size (i.e., time series length); for acronyms of variables, see S10 Table.

Model	R^2	ΔAICc	k	n
$\Delta S \sim \log(S_{t-1}) + \log(PP_W) + HAB + PER + PER\times\log(S_{t-1}) + T_{MIN} + \log(N)$	0.82	0	8	36
**$\Delta S \sim \log(S_{t-1}) + PP_W + HAB + PER + PER\times\log(S_{t-1}) + T_{MIN}$**	**0.80**	**0.2**	**7**	**36**
$\Delta S \sim \log(S_{t-1}) + PP_W + HAB + PER + PER\times\log(S_{t-1}) + T_{MIN} + \log(S_{t-2})$	0.85	1.0	8	34
$\Delta S \sim \log(S_{t-1}) + PP_W + HAB + PER + PER\times\log(S_{t-1}) + T_{MIN} + HAB\times T_{MIN}$	0.82	1.4	8	36
$\Delta S \sim \log(S_{t-1}) + \log(PP_W) + HAB + PER + PER\times\log(S_{t-1}) + T_{MIN}$	0.79	2.1	7	36
$\Delta S \sim \log(S_{t-1}) + \log(PP_W) + HAB + PER + PER\times\log(S_{t-1}) + T_{MIN}$	0.79	2.1	7	36
$\Delta S \sim \log(S_{t-1}) + \log(PP_W) + HAB + PER + PER\times\log(S_{t-1}) + T_{MIN} + \log(N) + \log(N)\times HAB$	0.83	2.4	9	36
$\Delta S \sim \log(S_{t-1}) + PP_W + HAB + PER + PER\times\log(S_{t-1})$	0.77	2.6	6	36
$\Delta S \sim \log(S_{t-1}) + \log(PP_W) + HAB + PER + PER\times\log(S_{t-1}) + T_{MIN} + \log(PP_W)\times HAB$	0.81	2.8	8	36
$\Delta S \sim \log(S_{t-1}) + PP_W + HAB + PER + PER\times\log(S_{t-1}) + PER\times HAB + T_{MIN}$	0.81	2.9	8	36
$\Delta S \sim \log(S_{t-1}) + \log(PP_W) + HAB + PER + PER\times\log(S_{t-1}) + T_{MIN} + \log(PP_W)\times PER$	0.81	3.2	8	36
$\Delta S \sim \log(S_{t-1}) + YR_{92} + PP_W + HAB + PER + PER\times\log(S_{t-1}) + T_{MIN}$	0.80	3.4	8	36
$\Delta S \sim \log(S_{t-1}) + YR_{92} + PP_W + HAB + PER + PER\times\log(S_{t-1}) + YR_{92}\times PER + T_{MIN}$	0.80	3.4	9	36
$\Delta S \sim \log(S_{t-1}) + PP_W + HAB + PER + PER\times\log(S_{t-1}) + T_{MEAN}$	0.78	3.5	7	36
$\Delta S \sim \log(S_{t-1}) + PP_W + HAB + PER + PER\times\log(S_{t-1}) + T_{MIN} + \log(S_{t-2}) + \log(S_{t-2})\times HAB$	0.85	3.8	9	34
$\Delta S \sim \log(S_{t-1}) + \log(PP_W) + HAB + PER + PER\times\log(S_{t-1}) + T_{MIN} + \log(PP_W)\times HAB + \log(PP_W)\times PER$	0.82	4.0	9	36
$\Delta S \sim \log(S_{t-1}) + PP_W + HAB + PER + PER\times\log(S_{t-1}) + PER\times PP_W$	0.78	4.0	7	36
$\Delta S \sim \log(S_{t-1}) + \log(PP_W) + HAB + PER + PER\times\log(S_{t-1}) + PER\times HAB + T_{MIN}$	0.80	5.0	8	36
$\Delta S \sim \log(S_{t-1}) + \log(PP_W) + HAB + PER + PER\times\log(S_{t-1}) + PER\times HAB$	0.77	5.1	7	36
$\Delta S \sim \log(S_{t-1}) + PP_W + HAB + PER + PER\times\log(S_{t-1}) + T_{MAX}$	0.77	5.2	7	36
$\Delta S \sim \log(S_{t-1}) + PP_W + HAB + PER + PER\times\log(S_{t-1}) + T_{MIN} + S_{t-2}$	0.83	5.3	7	34
$\Delta S \sim \log(S_{t-1}) + YR_{92} + PP_W + HAB + PER + PER\times\log(S_{t-1}) + YR_{92}\times HAB + T_{MIN}$	0.81	7.1	9	36
$\Delta S \sim \log(S_{t-1}) + YR_{92} + PP_W + HAB + PER + PER\times\log(S_{t-1}) + YR_{92}\times HAB + YR_{92}\times PER + T_{MIN}$	0.81	7.1	10	36
$\Delta S \sim \log(S_{t-1}) + PP_W + HAB + PER + PER\times\log(S_{t-1}) + T_{MIN} + S_{t-2} + S_{t-2}\times HAB$	0.84	7.5	9	34
$\Delta S \sim \log(S_{t-1}) + \log(PP_W) + HAB + PER + PER\times\log(S_{t-1}) + PER\times HAB$	0.76	7.5	7	36
$\Delta S \sim \log(S_{t-1}) + \log(PP_W) + PER + PER\times\log(S_{t-1}) + T_{MIN} + \log(PP_W)\times PER$	0.76	7.5	7	36
$\Delta S \sim \log(S_{t-1}) + PP_W + HAB + N + PER$	0.72	9.6	6	36
$\Delta S \sim \log(S_{t-1}) + PP_W + PER + PER\times\log(S_{t-1}) + T_{MIN} + \log(S_{t-2})$	0.78	9.7	7	34
$\Delta S \sim \log(S_{t-1}) + PP_W + HAB + N + N{:}PER$	0.71	10.8	6	36
$\Delta S \sim \log(S_{t-1}) + PP_W + HAB + PER$	0.68	11.3	5	36
