# Supplementary material for: Two decades of climate driving the dynamics of functional and taxonomic diversity of a tropical small mammal community in western Mexico
Source: PLoS One. 2017 Dec 11;12(12):e0189104. doi: 10.1371/journal.pone.0189104 (PMC5724848; doi:10.1371/journal.pone.0189104)
Supplement: S4 Table — Results for the 30 best-performing models (i.e., lowest AICc values) are shown; the selected model is highlighted in bold type. R2: determination coefficient, ΔAICc: difference between model’s AICc and the lowest AICc value, k: number of parameters fitted, n: sample size (i.e., time series length); for acronyms of variables, see S10 Table. (PDF) [file pone.0189104.s013.pdf]

S4 Table: Model selection for the dynamics of deviations of functional diversity (according to species' occurrence) from null model expectations in the dry season. Results for the 30 best-performing models (i.e., lowest AICc values) are shown; the selected model is highlighted in bold type. R^2: determination coefficient, ΔAICc: difference between model's AICc and the lowest AICc value, k: number of parameters fitted, n: sample size (i.e., time series length); for acronyms of variables, see S10 Table.

Models	R^2	ΔAICc	k	n
**ΔFDo ~ $dFDo_{t-1}$ + S_{t-1} + S_t + HAB + S×HAB + $dFDo_{t-1}$×HAB**	**0.69**	**0**	**7**	**34**
ΔFDo ~ $dFDo_{t-1}$ + S_{t-1} + S_t + HAB + S_t×HAB + $dFDo_{t-1}$×HAB + S_{t-1}×HAB	0.70	2.1	8	34
ΔFDo ~ $dFDo_{t-1}$ + S_{t-1} + S_t + HAB + S_t×HAB	0.62	3.4	6	34
ΔFDo ~ $dFDo_{t-1}$ + S_{t-1} + S_t + HAB + S_t×HAB + S_{t-1}×S_{t-2}	0.62	6.3	7	34
ΔFDo ~ $dFDo_{t-1}$ + S_{t-1} + S_t + HAB + S_t×HAB + S_{t-1}×HAB	0.62	6.8	7	34
ΔFDo ~ $dFDo_{t-1}$ + S_{t-1} + S_t + HAB + S_t×HAB + S_{t-1}×HAB	0.62	6.8	7	34
ΔFDo ~ $dFDo_{t-1}$ + S_t + PP_D + HAB + S_t×HAB	0.55	8.4	6	34
ΔFDo ~ $dFDo_{t-1}$ + S_t + HAB + S_t×HAB	0.51	8.5	5	34
ΔFDo ~ $dFDo_{t-1}$ + T_{MAX} + S_t + HAB + S_t×HAB	0.54	9.9	6	34
ΔFDo ~ $dFDo_{t-1}$ + HAB	0.38	10.6	3	34
ΔFDo ~ $dFDo_{t-1}$ + $dFDo_{t-2}$ + S_t + HAB + S_t×HAB	0.52	10.8	6	34
ΔFDo ~ $dFDo_{t-1}$ + S_t + PP_D + HAB + S_t×HAB + log(N)	0.57	10.9	7	34
ΔFDo ~ $dFDo_{t-1}$ + T_{MIN} + S_t + HAB + S_t×HAB	0.52	10.9	6	34
ΔFDo ~ $dFDo_{t-1}$ + S_t + HAB + S_t×HAB + log(N)	0.52	11.2	6	34
ΔFDo ~ $dFDo_{t-1}$ + T_{MEAN} + S_t + HAB + S_t×HAB	0.51	11.6	6	34
ΔFDo ~ $dFDo_{t-1}$ + S_t	0.35	12.4	3	34
ΔFDo ~ $dFDo_{t-1}$ + PP_D	0.34	12.6	3	34
ΔFDo ~ $dFDo_{t-1}$ + T_{MAX} + S_t + HAB + S_t×HAB + T_{MAX}×HAB	0.59	12.7	7	34
ΔFDo ~ $dFDo_{t-1}$ + S_t + HAB	0.38	13.4	4	34
ΔFDo ~ $dFDo_{t-1}$ + T_{MIN} + S_t + HAB + S_t×HAB + T_{MIN}×HAB	0.52	14.2	7	34
ΔFDo ~ $dFDo_{t-1}$ + $dFDo_{t-2}$ + S_t + HAB + S_t×HAB + $dFDo_{t-2}$×HAB	0.52	14.3	7	34
ΔFDo ~ $dFDo_{t-1}$ + S_t + PP_D + PP_W + HAB + S_t×HAB + log(N)	0.57	14.6	8	34
ΔFDo ~ $dFDo_{t-1}$ + PP_D + PP_W + HAB + S_t×HAB + log(N)	0.57	14.6	7	34
ΔFDo ~ $dFDo_{t-1}$ + S_t + PP_W + HAB + S_t×HAB + log(N)	0.52	14.7	7	34
ΔFDo ~ $dFDo_{t-1}$ + PP_D + HAB + log(N)	0.41	14.8	5	34
ΔFDo ~ $dFDo_{t-1}$ + S_t + PP_D	0.35	15.0	4	34
ΔFDo ~ $dFDo_{t-1}$ + T_{MEAN} + S_t + HAB + S_t×HAB + T_{MEAN}×HAB	0.51	15.1	7	34
ΔFDo ~ $dFDo_{t-1}$ + S_t + PP_D + PP_W + HAB + PP_D×HAB + S_t×HAB + log(N)	0.60	15.9	9	34
ΔFDo ~ $dFDo_{t-1}$ + S_t + PP_D + HAB	0.39	15.9	5	34
ΔFDo ~ $dFDo_{t-1}$ + S_t + PP_D + HAB + log(N)	0.43	16.8	6	34
