# Supplementary material for: Two decades of climate driving the dynamics of functional and taxonomic diversity of a tropical small mammal community in western Mexico
Source: PLoS One. 2017 Dec 11;12(12):e0189104. doi: 10.1371/journal.pone.0189104 (PMC5724848; doi:10.1371/journal.pone.0189104)
Supplement: S5 Table — Results for the 30 best-performing models (i.e., lowest AICc values) are shown; the selected model is highlighted in bold. R2: determination coefficient, ΔAICc: difference between model’s AICc and the lowest AICc value, k: number of parameters fitted, n: sample size (i.e., time series length); for acronyms of variables, see S10 Table. (PDF) [file pone.0189104.s014.pdf]

S5 Table: Model selection for the dynamics of deviations of functional diversity (according to species' occurrence) from null model expectations in the wet season. Results for the 30 best-performing models (i.e., lowest AICc values) are shown; the selected model is highlighted in bold. R^2: determination coefficient, ΔAICc: difference between model's AICc and the lowest AICc value, k: number of parameters fitted, n: sample size (i.e., time series length); for acronyms of variables, see S10 Table.

Model	R^2	ΔAICc	k	n
**ΔFDo ~ dFDo$_{t-1}$ + S$_{t-1}$**	**0.43**	**0**	**3**	**36**
ΔFDo ~ log(dFDo$_{t-1}$+1) + S$_{t-1}$	0.43	0.04	3	36
ΔFDo ~ dFDo$_{t-1}$ + log(S$_{t-1}$)	0.41	1.2	3	36
ΔFDo ~ dFDo$_{t-1}$ + S$_{t-1}$ + HAB	0.44	2.0	4	36
ΔFDo ~ dFDo$_{t-1}$ + S$_t$ + S$_{t-1}$ + HAB + dFDo$_{t-1}$×HAB	0.53	2.0	6	36
ΔFDo ~ dFDo$_{t-1}$ + S$_t$ + S$_{t-1}$ + HAB + dFDo$_{t-1}$×HAB + S$_t$×HAB	0.55	3.5	7	36
ΔFDo ~ dFDo$_{t-1}$ + S$_{t-1}$ + HAB + dFDo$_{t-1}$×HAB	0.46	3.9	5	36
ΔFDo ~ dFDo$_{t-1}$ + S$_t$ + S$_{t-1}$ + HAB + dFDo$_{t-1}$×HAB + S$_{t-1}$×HAB	0.54	4.6	8	36
ΔFDo ~ dFDo$_{t-1}$ + HAB + dFDo$_{t-1}$×HAB	0.39	4.9	4	36
ΔFDo ~ dFDo$_{t-1}$ + HAB	0.33	6.0	3	36
ΔFDo ~ dFDo$_{t-1}$ + S$_t$ + HAB + dFDo$_{t-1}$×HAB	0.42	6.5	5	36
ΔFDo ~ dFDo$_{t-1}$ + S$_{t-1}$ + HAB + dFDo$_{t-1}$×HAB + S$_{t-1}$×HAB	0.46	7.0	6	36
ΔFDo ~ dFDo$_{t-1}$ + S$_t$ + S$_{t-1}$ + HAB + dFDo$_{t-1}$×HAB + S$_t$×HAB + S$_{t-1}$×HAB	0.55	7.0	8	36
ΔFDo ~ dFDo$_{t-1}$ + T$_{MIN}$ + HAB + dFDo$_{t-1}$×HAB	0.40	7.3	5	36
ΔFDo ~ dFDo$_{t-1}$ + T$_{MEAN}$ + HAB + dFDo$_{t-1}$×HAB	0.40	7.5	5	36
ΔFDo ~ dFDo$_{t-1}$ + T$_{MAX}$ + HAB + dFDo$_{t-1}$×HAB	0.40	7.7	5	36
ΔFDo ~ dFDo$_{t-1}$ + S$_t$ + HAB + dFDo$_{t-1}$×HAB + S$_t$×HAB	0.44	7.7	6	36
ΔFDo ~ dFDo$_{t-1}$ + PP$_D$ + HAB	0.34	7.9	4	36
ΔFDo ~ dFDo$_{t-1}$ + PP$_W$ + HAB	0.33	8.5	4	36
ΔFDo ~ dFDo$_{t-1}$	0.21	9.1	2	36
ΔDFo ~ dFDo$_{t-1}$ + T$_{MAX}$ + HAB + dFDo$_{t-1}$×HAB + T$_{MAX}$×HAB	0.42	9.6	6	36
ΔFDo ~ dFDo$_{t-1}$ + T$_{MEAN}$ + HAB + dFDo$_{t-1}$×HAB + T$_{MEAN}$×HAB	0.41	10.0	6	36
ΔFDo ~ dFDo$_{t-1}$ + PP$_D$ + PP$_W$ + HAB	0.35	10.4	5	36
ΔFDo ~ dFDo$_{t-1}$ + T$_{MIN}$ + HAB + dFDo$_{t-1}$×HAB + T$_{MIN}$×HAB	0.40	10.4	6	36
ΔFDo ~ dFDo$_{t-1}$ + S$_t$ + PP$_D$ + HAB	0.34	10.8	5	36
ΔFDo ~ dFDo$_{t-1}$ + S$_t$ + PP$_W$ + HAB	0.34	11.0	5	36
ΔFDo ~ dFDo$_{t-1}$ + PP$_D$	0.22	11.2	3	36
ΔFDo ~ dFDo$_{t-1}$ + S$_t$ + PP$_D$ + PP$_W$ + HAB	0.35	13.3	6	36
ΔFDo ~ dFDo$_{t-1}$ + PP$_D$ + PP$_W$ + HAB + log(N)	0.35	13.5	6	36
ΔFDo ~ dFDo$_{t-1}$ + PP$_D$ + PP$_W$	0.23	13.6	4	36
