# Supplementary material for: Two decades of climate driving the dynamics of functional and taxonomic diversity of a tropical small mammal community in western Mexico
Source: PLoS One. 2017 Dec 11;12(12):e0189104. doi: 10.1371/journal.pone.0189104 (PMC5724848; doi:10.1371/journal.pone.0189104)
Supplement: S6 Table — Results for the 30 best-performing models (i.e., lowest AICc values) are shown; the selected model is highlighted in bold. R2: determination coefficient, ΔAICc: difference between model’s AICc and the lowest AICc value, k: number of parameters fitted, n: sample size (i.e., time series length); for acronyms of variables, see S10 Table. (PDF) [file pone.0189104.s015.pdf]

S6 Table: Model selection for the dynamics of deviations of functional diversity (according to number of individuals) from null model expectations in the dry season. Results for the 30 best-performing models (i.e., lowest AICc values) are shown; the selected model is highlighted in bold. R^2: determination coefficient, ΔAICc: difference between model's AICc and the lowest AICc value, k: number of parameters fitted, n: sample size (i.e., time series length); for acronyms of variables, see S10 Table.

Model	R^2	ΔAICc	k	n
**$\Delta FDn \sim dFDn_{t-1} + S_t + HAB + \log(N)$**	**0.74**	**0**	**5**	**34**
$\Delta FDn \sim dFDn_{t-1} + S_t + dFDn_{t-2} + HAB + \log(N) + dFDn_{t-2} \times HAB$	0.79	0.2	7	34
$\Delta FDn \sim dFDn_{t-1} + S_t + T_{MIN} + HAB + \log(N)$	0.76	0.3	6	34
$\Delta FDn \sim dFDn_{t-1} + S_t + T_{MEAN} + HAB + \log(N)$	0.76	0.9	6	34
$\Delta FDn \sim dFDn_{t-1} + S + PP_W + HAB + \log(N)$	0.75	1.6	6	34
$\Delta FDn \sim dFDn_{t-1} + S_t + T_{MAX} + HAB + \log(N)$	0.75	2.2	6	34
$\Delta FDn \sim dFDn_{t-1} + S_t + S_{t-1} + HAB + \log(N)$	0.75	2.3	6	34
$\Delta FDn \sim dFDn_{t-1} + S_t + HAB + \log(N) + dFDn_{t-1} \times HAB$	0.75	2.8	6	34
$\Delta FDn \sim dFDn_{t-1} + S_t + HAB + \log(N) + dFDn_{t-1} \times HAB$	0.75	2.8	6	34
$\Delta FDn \sim dFDn_{t-1} + S_t + dFDn_{t-2} + HAB + \log(N)$	0.74	3.1	6	34
$\Delta FDn \sim dFDn_{t-1} + S_t + PP_D + HAB + \log(N)$	0.74	3.1	6	34
$\Delta FDn \sim dFDn_{t-1} + S_t + HAB + \log(N) + S \times HAB$	0.74	3.2	6	34
$\Delta FDn \sim dFDn_{t-1} + S_t + \log(N)$	0.69	3.4	4	34
$\Delta FDn \sim dFDn_{t-1} + S_t + T_{MIN} + HAB + \log(N) + T_{MIN} \times HAB$	0.77	3.6	7	34
$\Delta FDn \sim dFDn_{t-1} + S_t + T_{MEAN} + HAB + \log(N) + T_{MEAN} \times HAB$	0.76	3.8	7	34
$\Delta FDn \sim dFDn_{t-1} + S_t + T_{MAX} + HAB + \log(N) + T_{MAX} \times HAB$	0.76	4.6	7	34
$\Delta FDn \sim dFDn_{t-1} + S_t + PP_D + PP_W + HAB + \log(N)$	0.76	4.9	7	34
$\Delta FDn \sim dFDn_{t-1} + S_t + S_{t-1} + HAB + \log(N) + S_{t-1} \times HAB$	0.75	5.7	7	34
$\Delta FDn \sim dFDn_{t-1} + S_t + HAB + \log(N) + dFDn_{t-1} \times HAB + S \times HAB$	0.75	6.2	7	34
$\Delta FDn \sim dFDn_{t-1} + S_t + PP_W + \log(N)$	0.69	6.4	5	34
$\Delta FDn \sim dFDn_{t-1} + S_t + PP_D + PP_W + HAB + PP_D \times HAB + \log(N)$	0.76	7.6	8	34
$\Delta FDn \sim dFDn_{t-1} + S_t + PP_D + PP_W + HAB + dFDn_{t-1} \times HAB + \log(N)$	0.76	8.3	8	34
$\Delta FDn \sim dFDn_{t-1} + S_t + PP_D + PP_W + HAB + S_t \times HAB + \log(N)$	0.76	8.5	8	34
$\Delta FDn \sim dFDn_{t-1} + S_t + PP_D + PP_W + HAB + PP_W \times HAB + \log(N)$	0.76	8.6	8	34
$\Delta FDn \sim dFDn_{t-1} + S_t + PP_D + PP_W + \log(N)$	0.69	9.3	6	34
$\Delta FDn \sim dFDn_{t-1} + PP_W + HAB + \log(N)$	0.66	9.5	5	34
$\Delta FDn \sim dFDn_{t-1} + PP_D + PP_W + HAB + \log(N)$	0.68	10.8	6	34
$\Delta FDn \sim dFDn_{t-1} + S_t + PP_D + PP_W + HAB + dFDn_{t-1} \times HAB + PP_D \times HAB + \log(N)$	0.77	11.3	9	34
$\Delta FDn \sim dFDn_{t-1} + S_t + PP_D + PP_W + HAB + PP_D \times HAB + PP_W \times HAB + \log(N)$	0.76	11.6	9	34
$\Delta FDn \sim dFDn_{t-1} + S_t + PP_D + PP_W + HAB + PP_D \times HAB + S \times HAB + \log(N)$	0.76	11.6	9	34
