# Supplementary material for: Two decades of climate driving the dynamics of functional and taxonomic diversity of a tropical small mammal community in western Mexico
Source: PLoS One. 2017 Dec 11;12(12):e0189104. doi: 10.1371/journal.pone.0189104 (PMC5724848; doi:10.1371/journal.pone.0189104)
Supplement: S7 Table — Results for the 30 best-performing models (i.e., lowest AICc values) are shown; the selected model is highlighted in bold. R2: determination coefficient, ΔAICc: difference between model's AICc and the lowest AICc value, k: number of parameters fitted, n: sample size (i.e., time series length); for acronyms of variables, see S10 Table. (PDF) [file pone.0189104.s016.pdf]

S7 Table: **Model selection for the dynamics of deviations of functional diversity (according to number of individuals) from null model expectations in the wet season.** Results for the 30 best-performing models (i.e., lowest AICc values) are shown; the selected model is highlighted in bold. R^2: determination coefficient, ΔAICc: difference between model's AICc and the lowest AICc value, k: number of parameters fitted, n: sample size (i.e., time series length); for acronyms of variables, see S10 Table.

Model	R^2	ΔAICc	k	n
$\Delta FDn \sim dFDn_{t-1} + T_{MIN} + HAB + \log(N) + dFDn_{t-1}\times HAB$	0.62	0	6	36
**$\Delta FDn \sim dFDn_{t-1} + HAB + \log(N) + dFDn_{t-1}\times HAB$**	**0.58**	**0.1**	**5**	**36**
$\Delta FDn \sim dFDn_{t-1} + T_{MEAN} + HAB + \log(N) + dFDn_{t-1}\times HAB$	0.61	0.4	6	36
$\Delta FDn \sim dFDn_{t-1} + T_{MAX} + HAB + \log(N) + dFDn_{t-1}\times HAB$	0.60	1.3	6	36
$\Delta FDn \sim \log(dFDn_{t-1}+1) + HAB + \log(N) + dFDn_{t-1}\times HAB$	0.60	2.1	5	36
$\Delta FDn \sim dFDn_{t-1} + S_t + HAB + \log(N) + dFDn_{t-1}\times HAB$	0.59	2.4	6	36
$\Delta FDn \sim dFDn_{t-1} + S_t + HAB + \log(N) + dFDn_{t-1}\times HAB$	0.59	2.4	6	36
$\Delta FDn \sim dFDn_{t-1} + PP_W + HAB + \log(N)$	0.55	2.7	5	36
$\Delta FDn \sim dFDn_{t-1} + PP_W + \log(N)$	0.51	3.0	4	36
$\Delta FDn \sim dFDn_{t-1} + S_{t-1} + HAB + \log(N) + dFDn_{t-1}\times HAB$	0.58	3.1	6	36
$\Delta FDn \sim dFDn_{t-1} + S_t + HAB + \log(N) + dFDn_{t-1}\times HAB + S_t\times HAB$	0.62	3.1	7	36
$\Delta FDn \sim dFDn_{t-1} + HAB + \log(N)$	0.51	3.2	4	36
$\Delta FDn \sim \log(dFDn_{t-1}+1) + HAB + \log(N)$	0.51	3.2	4	36
$\Delta FDn \sim dFDn_{t-1} + T_{MIN} + HAB + \log(N) + dFDn_{t-1}\times HAB + T_{MIN}\times HAB$	0.62	3.3	7	36
$\Delta FDn \sim dFDn_{t-1} + T_{MEAN} + HAB + \log(N) + dFDn_{t-1}\times HAB + T_{MEAN}\times HAB$	0.62	3.6	7	36
$\Delta FDn \sim dFDn_{t-1} + \log(N)$	0.46	4.1	3	36
$\Delta FDn \sim \log(dFDn_{t-1}+1) + \log(N)$	0.45	4.3	3	36
$\Delta FDn \sim dFDn_{t-1} + T_{MAX} + HAB + \log(N) + dFDn_{t-1}\times HAB + T_{MAX}\times HAB$	0.61	4.3	7	36
$\Delta FDn \sim dFDn_{t-1} + PP_D + HAB + \log(N)$	0.53	4.8	5	36
$\Delta FDn \sim dFDn_{t-1} + PP_D + PP_W + HAB + \log(N)$	0.56	5.0	6	36
$\Delta FDn \sim dFDn_{t-1} + S_t + HAB + \log(N)$	0.52	5.2	5	36
$\Delta FDn \sim dFDn_{t-1} + PP_W + \log(N) + dFDn_{t-1}:PP_W$	0.51	5.6	5	36
$\Delta FDn \sim dFDn_{t-1} + PP_D + PP_W + \log(N)$	0.51	5.7	5	36
$\Delta FDn \sim dFDn_{t-1} + S_t + PP_W + HAB + \log(N)$	0.55	5.7	6	36
$\Delta FDn \sim dFDn_{t-1} + S_{t-1} + HAB + \log(N) + dFDn_{t-1}\times HAB + S_{t-1}\times HAB$	0.59	6.2	7	36
$\Delta FDn \sim dFDn_{t-1} + dFDn_{t-1}^2 + \log(N)$	0.46	6.3	3	36
$\Delta FDn \sim dFDn_{t-1} + PP_W + HAB$	0.45	7.0	4	36
$\Delta FDn \sim dFDn_{t-1} + S_t + PP_D + HAB + \log(N)$	0.53	7.4	6	36
$\Delta FDn \sim dFDn_{t-1} + S_t + PP_D + PP_W + HAB + S\times HAB + \log(N)$	0.61	7.6	8	36
$\Delta FDn \sim dFDn_{t-1} + S_t + PP_D + PP_W + HAB + dFDn_{t-1}\times HAB + \log(N)$	0.61	7.7	8	36
