# Supplementary material for: Two decades of climate driving the dynamics of functional and taxonomic diversity of a tropical small mammal community in western Mexico
Source: PLoS One. 2017 Dec 11;12(12):e0189104. doi: 10.1371/journal.pone.0189104 (PMC5724848; doi:10.1371/journal.pone.0189104)
Supplement: S8 Table — Results for the 30 best-performing models (i.e., lowest AICc values) are shown; the selected model is highlighted in bold. R2: determination coefficient, ΔAICc: difference between model’s AICc and the lowest AICc value, k: number of parameters fitted, n: sample size (i.e., time series length); for acronyms of variables, see S10 Table. (PDF) [file pone.0189104.s017.pdf]

S8 Table: Model selection for the dynamic of deviations of functional diversity (according to biomass) from null model expectations in the dry season. Results for the 30 best-performing models (i.e., lowest AICc values) are shown; the selected model is highlighted in bold. R^2: determination coefficient, ΔAICc: difference between model's AICc and the lowest AICc value, k: number of parameters fitted, n: sample size (i.e., time series length); for acronyms of variables, see S10 Table.

Model	R2	ΔAICc	k	n
**$\Delta FDw \sim dFDw_{t-1} + YR + YR^2$**	**0.57**	**0**	**4**	**34**
$\Delta FDw \sim e^{dFDwt-1} + YR$	0.53	0.1	3	34
$\Delta FDw \sim dFDw_{t-1} + e^{YR/1000}$	0.53	0.3	3	34
$\Delta FDw \sim dFDw_{t-1} + YR$	0.53	0.3	3	34
$\Delta FDw \sim dFDw_{t-1} + \log(YR)$	0.53	0.3	3	34
$\Delta FDw \sim \log(dFDw_{t-1}+1) + YR$	0.52	0.5	3	34
$\Delta FDw \sim \log(dFDw_{t-1}+1) + \log(T_{MIN}) + YR$	0.54	1.8	4	34
$\Delta FDw \sim \log(dFDw_{t-1}+1) + T_{MIN} + YR$	0.54	1.9	4	34
$\Delta FDw \sim dFDw_{t-1} + YR + HAB$	0.54	1.9	4	34
$\Delta FDw \sim dFDw_{t-1} + YR + \log(N)$	0.54	2.1	4	34
$\Delta FDw \sim dFDw_{t-1} + YR + YR^2 + \log(N)$	0.57	2.8	5	34
$\Delta FDw \sim \log(dFDw_{t-1}+1) + T_{MIN} + YR + YR \times HAB$	0.56	3.8	5	34
$\Delta FDw \sim dFDw_{t-1} + T_{MIN}$	0.46	4.7	3	34
$\Delta FDw \sim \log(dFDw_{t-1}+1) + T_{MIN}$	0.46	4.8	3	34
$\Delta FDw \sim dFDw_{t-1} + YR + HAB + YR \times HAB$	0.54	4.8	5	34
$\Delta FDw \sim dFDw_{t-1} + YR + HAB + YR \times HAB$	0.54	4.8	5	34
$\Delta FDw \sim \log(dFDw_{t-1}+1) + YR + HAB + YR \times HAB$	0.54	5.0	5	34
$\Delta FDw \sim dFDw_{t-1} + YR + YR^2 + HAB + YR \times HAB$	0.58	5.0	6	34
$\Delta FDw \sim dFDw_{t-1} + TMEAN$	0.45	5.4	3	34
$\Delta FDw \sim dFDw_{t-1} + S_t$	0.45	5.8	3	34
$\Delta FDw \sim \log(dFDw_{t-1}+1)$	0.40	6.0	2	34
$\Delta FDw \sim dFDw_{t-1}$	0.40	6.0	2	34
$\Delta FDw \sim dFDw_{t-1} + dFDw_{t-2}$	0.44	6.3	3	34
$\Delta FDw \sim dFDw_{t-1} + T_{MIN} + HAB$	0.47	6.9	4	34
$\Delta FDw \sim dFDw_{t-1} + T_{MAX}$	0.42	7.2	3	34
$\Delta FDw \sim dFDw_{t-1} + T_{MEAN} + HAB$	0.46	7.5	4	34
$\Delta FDw \sim dFDw_{t-1} + S_t + PP_D$	0.46	7.8	4	34
$\Delta FDw \sim dFDw_{t-1} + T_{MIN} + HAB + T_{MIN} \times HAB$	0.50	7.9	5	34
$\Delta FDw \sim dFDw_{t-1} + HAB$	0.41	8.0	3	34
$\Delta FDw \sim dFDw_{t-1} + S_t + \log(N)$	0.45	8.0	4	34
