# Supplementary material for: Two decades of climate driving the dynamics of functional and taxonomic diversity of a tropical small mammal community in western Mexico
Source: PLoS One. 2017 Dec 11;12(12):e0189104. doi: 10.1371/journal.pone.0189104 (PMC5724848; doi:10.1371/journal.pone.0189104)
Supplement: S9 Table — Results for the 30 best-performing models (i.e., lowest AICc values) are shown; the selected model is highlighted in bold. R2: determination coefficient, ΔAICc: difference between model’s AICc and the lowest AICc value, k: number of parameters fitted, n: sample size (i.e., time series length); for acronyms of variables, see S10 Table. (PDF) [file pone.0189104.s018.pdf]

S9 Table: Model selection for the dynamic of deviations of functional diversity (according to biomass) from null model expectations in the wet season. Results for the 30 best-performing models (i.e., lowest AICc values) are shown; the selected model is highlighted in bold. R^2: determination coefficient, ΔAICc: difference between model's AICc and the lowest AICc value, k: number of parameters fitted, n: sample size (i.e., time series length); for acronyms of variables, see S10 Table.

Model	R^2	ΔAICc	k	N
**ΔFDw ~ $dFDw_{t-1}$ + S_{t-1} + HAB + log(N) + S_{t-1}×HAB**	**0.78**	**0**	**6**	**36**
ΔFDw ~ $dFDw_{t-1}$ + T_{MEAN} + HAB + log(N) + T_{MEAN}×HAB	0.77	1.8	6	36
ΔFDw ~ log($dFDw_{t-1}$+1) + S_{t-1} + HAB + log(N) + S_{t-1}×HAB	0.77	1.9	6	36
ΔFDw ~ $dFDw_{t-1}$ + T_{MEAN} + log(N)	0.73	2.0	4	36
ΔFDw ~ log($dFDw_{t-1}$+1) + S_{t-1} + HAB + log(N)	0.75	2.1	5	36
ΔFDw ~ $dFDw_{t-1}$ + T_{MAX} + log(N)	0.72	2.4	4	36
ΔFDw ~ $dFDw_{t-1}$ + YR + S_{t-1} + HAB + log(N) + S_{t-1}×HAB	0.78	2.8	7	36
ΔFDw ~ $dFDw_{t-1}$ + S_{t-1} + HAB + YR + log(N) + S_{t-1}×HAB	0.78	2.8	7	36
ΔFDw ~ $dFDw_{t-1}$ + T_{MIN} + HAB + log(N) + T_{MIN}×HAB	0.76	3.4	6	36
ΔFDw ~ $dFDw_{t-1}$ + log(N)	0.69	3.5	3	36
ΔFDw ~ $dFDw_{t-1}$ + PP_D + log(N)	0.71	3.6	4	36
ΔFDw ~ $dFDw_{t-1}$ + HAB + log(N)	0.71	3.6	4	36
ΔFDw ~ $dFDw_{t-1}$ + T_{MAX} + HAB + log(N) + T_{MAX}×HAB	0.76	3.7	6	36
ΔFDw ~ $dFDw_{t-1}$ + T_{MIN} + log(N)	0.71	3.8	4	36
ΔFDw ~ $dFDw_{t-1}$ + PP_D + HAB + log(N)	0.73	3.9	5	36
ΔFDw ~ $dFDw_{t-1}$ + YR + S_{t-1} + HAB + log(N) + S_{t-1}:HAB + YR×HAB	0.80	4.4	8	36
ΔFDw ~ $dFDw_{t-1}$ + S_{t-1} + HAB + YR + YR^2 + log(N) + S_{t-1}×HAB	0.79	4.6	8	36
ΔFDw ~ log($dFDw_{t-1}$+1) + S_{t-1} + HAB + YR + log(N) + S_{t-1}×HAB	0.77	4.6	7	36
ΔFDw ~ $dFDw_{t-1}$ + S_t + PP_D + HAB + log(N)	0.75	5.2	6	36
ΔFDw ~ $dFDw_{t-1}$ + HAB + log(N) + $dFDw_{t-1}$×HAB	0.72	5.2	5	36
ΔFDw ~ $dFDw_{t-1}$ + S_{t-1} + log(N)	0.70	5.3	4	36
ΔFDw ~ $dFDw_{t-1}$ + S_t + HAB + log(N)	0.72	5.5	5	36
ΔFDw ~ $dFDw_{t-1}$ + PP_D + PP_W + HAB + log(N)	0.74	6.2	6	36
ΔFDw ~ $dFDw_{t-1}$ + HAB + PP_D + log(N) + PP_D×HAB	0.74	6.4	6	36
ΔFDw ~ $dFDw_{t-1}$ + S_t + PP_D + log(N)	0.71	6.5	5	36
ΔFDw ~ log($dFDw_{t-1}$+1) + S_{t-1} + log(N)	0.69	6.6	4	36
ΔFDw ~ $dFDw_{t-1}$ + HAB + S_t + log(N) + S_t×HAB	0.73	7.9	6	36
ΔFDw ~ $dFDw_{t-1}$ + S_t + PP_D + PP_W + HAB + log(N)	0.75	8.3	7	36
ΔFDw ~ $dFDw_{t-1}$ + S_t + PP_W + HAB + log(N)	0.72	8.5	6	36
ΔFDw ~ $dFDw_{t-1}$ + S_t + PP_D + PP_W + log(N)	0.72	8.9	6	36
