# Supplementary material for: Two decades of climate driving the dynamics of functional and taxonomic diversity of a tropical small mammal community in western Mexico
Source: PLoS One. 2017 Dec 11;12(12):e0189104. doi: 10.1371/journal.pone.0189104 (PMC5724848; doi:10.1371/journal.pone.0189104)
Supplement: S10 Table — (PDF) [file pone.0189104.s019.pdf]

S10 Table: Acronyms and short description of variables in Tables S2 to S9.

Acronym	Variable description
ΔS	Between-seasons rate of change in species richness: $\log(S_t/S_{t-1})$.
S_t	Species richness of the season.
S_{t-1}	Species richness one season before (i.e. for the wet season corresponds to the previous dry season, and vice versa).
S_{t-2}	Species richness two seasons before (i.e. for the wet and dry season corresponds to the wet and dry season one year before, respectively).
ΔFDo	Between-seasons rate of change in deviations of functional diversity (according to species occurrence) from null-model expectations (dFDo): $dFDo_t$ - $dFDo_{t-1}$.
$dFDo_{t-1}$	dFDo value one season before (i.e. for the wet season corresponds to the previous dry season, and vice versa).
$dFDo_{t-2}$	dFDo value two seasons before (i.e. for the wet and dry season corresponds to the wet and dry season one year before, respectively).
ΔFDn	Between-seasons rate of change in deviations of functional diversity (according to nº of individuals of each species captured) from null-model expectations (dFDn): $dFDn_t$ - $dFDn_{t-1}$
$dFDn_{t-1}$	dFDn value one season before (i.e. for the wet season corresponds to the previous dry season, and vice versa).
$dFDn_{t-2}$	dFDn value two seasons before (i.e. for the wet and dry season corresponds to the wet and dry season one year before, respectively).
ΔFDw	Between-seasons rate of change in deviations of functional diversity (according to total biomass of individuals of each species captured) from null-model expectations (dFDw): $dFDw_t$ - $dFDw_{t-1}$
$dFDw_{t-1}$	dFDw value one season before (i.e. for the wet season corresponds to the previous dry season, and vice versa).
$dFDw_{t-2}$	dFDw value two seasons before (i.e. for the wet and dry season corresponds to the wet and dry season one year before, respectively).
HAB	Habitat; i.e. forest type: Upland Forest or Arroyo Forest
N	Total number of individual captured during the trapping sessions (i.e. sample size for community parameters estimation).
PP_W	Precipitation volume (mm) accumulated during the wet season; this value corresponds to the value of season for wet-season models, and of the previous season for dry-season models.
PP_D	Precipitation volume (mm) accumulated during the dry season; this value corresponds to the value of season for dry-season models, and of the previous season for wet-season models.
YR	Year, used to control for long-term temporal trends.
PER	Subdivision of the entire study period used to model species richness in the wet season due to an apparent regimen change between the 1990-1997 and the 1998-2007 periods.
YR_{92}	Dummy variable used to account for potential effects of an abnormally rainy dry season in 1992, which were not explained for the other variables in the database (including PP_D).
T_{MEAN}	Mean (grand-mean) temperature (ºC) of the season.
T_{MIN}	Mean minimum temperature for the season.
T_{MAX}	Mean maximum temperature for the season.
