# Supplementary material for: Two decades of climate driving the dynamics of functional and taxonomic diversity of a tropical small mammal community in western Mexico
Source: PLoS One. 2017 Dec 11;12(12):e0189104. doi: 10.1371/journal.pone.0189104 (PMC5724848; doi:10.1371/journal.pone.0189104)
Supplement: S11 Table — The traits on every category (habitat, habitat (stratum) use, diet and temporal activity) are multivariate variables standardized so the sum of the variables on every category sum 1. Habitat was obtained from IUCN (and its category numbers are included) [72], proportions of use were obtained from compilations, just as the variables in the other three categories [61,70,71,83]. Abbreviations are: Bm (Baiomys musculus), Lp (Liomys pictus), Mg (Megasorex gigas), Ns (Nyctomys sumichrasti), Oc (Oryzomys mexicanus), Om (Oryzomys melanotis), Ob (Osgoodomys banderanus, Pp (Peromyscus perfulvus), Rf (Reithrodontomys fulvescens), Sm (Sigmodon mascotensis), Sp (Spilogale pygmaea), Tc (Tlacuatzin canescens), Xn (Xenomys nelsoni). (PDF) [file pone.0189104.s020.pdf]

S11 Table. Functional traits database. The traits on every category (habitat, habitat (stratum) use, diet and temporal activity) are multivariate variables standardized so the sum of the variables on every category sum 1. Habitat was obtained from IUCN (and its category numbers are included) [1], proportions of use were obtained from compilations, just as the variables in the other three categories [2–5]. Abbreviations are: Bm (*Baiomys musculus), Lp (Liomys pictus), Mg (Megasorex gigas), Ns (Nyctomys sumichrasti), Oc (Oryzomys mexicanus), Om (Oryzomys melanotis), Ob (Osgoodomys banderanus, Pp (Peromyscus perfulvus), Rf (Reithrodontomys fulvescens), Sm (Sigmodon mascotensis), Sp (Spilogale pygmaea), Tc (Tlacuatzin canescens), Xn (Xenomys nelsoni).*

Spp	Habitat								Habitat (stratum) use					Diet				Temporal activity		
	Upland forest (1.5)	Arroyo forest (1.6)	forest (1)	Shrubland(3)	Grassland (4)	Artificial/terrestrial (14)	Wetlands (5)	Marine coastal/supratidal (14)	Terrestrial	Fossorial	Semi arboreal	Arboreal	Semi aquatic	Seed eating	Herbivorous	Insectivorous	Carnivorous	Daylight	Night	Twilight
Bm	0.182	0.091	0.182	0.182	0.182	0.182	0.000	0.000	1.000	0.000	0.000	0.000	0.000	0.313	0.625	0.063	0.000	0.000	0.500	0.500
Lp	0.250	0.250	0.250	0.250	0.000	0.000	0.000	0.000	1.000	0.000	0.000	0.000	0.000	0.833	0.083	0.083	0.000	0.000	0.500	0.500
Mg	0.000	0.000	0.333	0.333	0.000	0.333	0.000	0.000	0.625	0.313	0.000	0.000	0.063	0.000	0.000	1.000	0.000	1.000	0.000	0.000
Ns	0.200	0.400	0.400	0.000	0.000	0.000	0.000	0.000	0.063	0.000	0.625	0.313	0.000	0.313	0.625	0.063	0.000	0.000	0.500	0.500
Oc	0.333	0.333	0.000	0.000	0.000	0.000	0.333	0.000	0.385	0.000	0.192	0.038	0.385	0.250	0.250	0.250	0.250	0.000	0.500	0.500
Om	0.333	0.333	0.333	0.000	0.000	0.000	0.000	0.000	0.385	0.000	0.192	0.038	0.385	0.250	0.250	0.250	0.250	0.000	0.500	0.500
Ob	0.200	0.400	0.400	0.000	0.000	0.000	0.000	0.000	0.048	0.000	0.476	0.476	0.000	0.313	0.625	0.063	0.000	0.000	0.500	0.500
Pp	0.143	0.286	0.286	0.000	0.000	0.286	0.000	0.000	0.200	0.000	0.400	0.400	0.000	0.250	0.500	0.250	0.000	0.000	0.500	0.500
Rf	0.143	0.286	0.000	0.286	0.286	0.000	0.000	0.000	0.625	0.000	0.313	0.063	0.000	0.313	0.625	0.063	0.000	0.000	0.500	0.500
Sm	0.000	0.000	0.500	0.000	0.000	0.500	0.000	0.000	1.000	0.000	0.000	0.000	0.000	0.476	0.476	0.048	0.000	0.000	0.500	0.500
Sp	0.000	0.000	0.250	0.250	0.000	0.250	0.000	0.250	1.000	0.000	0.000	0.000	0.000	0.000	0.063	0.625	0.313	0.100	0.400	0.500
Tc	0.000	0.000	0.500	0.500	0.000	0.000	0.000	0.000	0.048	0.000	0.476	0.476	0.000	0.000	0.091	0.909	0.000	0.000	0.500	0.500
Xn	0.667	0.333	0.000	0.000	0.000	0.000	0.000	0.000	0.048	0.000	0.476	0.476	0.000	0.313	0.625	0.063	0.000	0.000	0.500	0.500
