# Supplementary material for: Two decades of climate driving the dynamics of functional and taxonomic diversity of a tropical small mammal community in western Mexico
Source: PLoS One. 2017 Dec 11;12(12):e0189104. doi: 10.1371/journal.pone.0189104 (PMC5724848; doi:10.1371/journal.pone.0189104)
Supplement: S12 Table — Abbreviations are: Bm (Baiomys musculus), Lp (Liomys pictus), Mg (Megasorex gigas), Ns (Nyctomys sumichrasti), Oc (Oryzomys mexicanus), Om (Oryzomys melanotis), Ob (Osgoodomys banderanus, Pp (Peromyscus perfulvus), Rf (Reithrodontomys fulvescens), Sm (Sigmodon mascotensis), Sp (Spilogale pygmaea), Tc (Tlacuatzin canescens), Xn (Xenomys nelsoni). D (Dry season), W (wet season). (PDF) [file pone.0189104.s021.pdf]

S12 Table. Abundance database upland forest. Abbreviations are: Bm (*Baiomys musculus), Lp (Liomys pictus), Mg (Megasorex gigas), Ns (Nyctomys sumichrasti), Oc (Oryzomys mexicanus), Om (Oryzomys melanotis), Ob (Osgoodomys banderanus, Pp (Peromyscus perfulvus), Rf (Reithrodontomys fulvescens), Sm (Sigmodon mascotensis), Sp (Spilogale pygmaea), Tc (Tlacuatzin canescens), Xn (Xenomys nelsoni). D (Dry season), W (wet season).*

Spp	1990		1991		1992		1993		1994		1995		1996		1997		1998		1999		2000		2001		2002		2003		2004		2005		2006		2007		2012	
	D	W	D	W	D	W	D	W	D	W	D	W	D	W	D	W	D	W	D	W	D	W	D	W	D	W	D	W	D	W	D	W	D	W	D	W	D	W
Bm	0	0	0	0	0	0	0	0	6	1	0	1	7	0	0	0	0	0	0	0	0	0	0	0	0	0	0	0	4	4	0	0	0	0	3	2	0	0
Lp	336	633	371	250	338	338	0	0	0	669	193	202	345	282	232	427	357	274	244	244	54	84	276	198	111	130	219	237	366	332	188	66	89	102	134	296	156	156
Mg	0	0	0	0	0	0	0	0	0	0	0	0	0	0	0	0	0	0	0	0	0	0	0	0	0	0	0	0	0	0	0	0	0	0	0	0	0	0
Ns	0	0	0	0	1	2	0	0	0	0	0	0	0	0	0	0	0	0	0	0	2	4	0	0	0	0	0	0	0	0	0	0	0	0	0	0	0	0
Oc	2	1	1	0	0	0	0	0	0	10	0	0	0	1	12	5	0	0	1	1	17	4	0	1	0	0	0	0	8	19	1	0	0	0	0	0	7	13
Om	2	2	0	0	0	0	0	0	0	0	0	5	0	0	14	1	3	0	0	0	11	0	0	0	0	0	0	0	0	1	0	0	0	0	0	0	7	9
Ob	1	1	0	0	0	3	0	0	0	0	6	3	21	9	16	15	14	4	4	0	81	16	4	0	2	3	4	3	16	19	19	0	0	0	0	0	4	1
Pp	0	0	0	0	0	0	0	0	0	0	0	0	4	2	2	1	0	0	0	0	44	16	2	0	0	0	0	0	8	1	0	1	0	0	0	0	8	0
Rf	0	0	5	1	6	5	31	19	0	13	6	7	9	2	17	11	1	0	0	0	0	0	0	0	0	0	0	1	0	0	0	1	0	0	0	0	0	2
Sm	0	0	0	0	0	0	0	0	0	1	4	14	2	0	0	0	0	3	0	0	0	0	0	0	0	0	0	0	9	19	0	2	0	0	0	0	0	0
Sp	0	1	0	1	0	1	0	0	0	1	0	0	0	1	0	3	0	0	0	0	0	0	0	0	0	0	0	0	0	0	0	0	0	0	1	0	0	0
Tc	1	0	4	5	18	2	1	1	0	5	0	0	0	0	10	4	1	0	2	0	0	0	1	0	0	0	0	0	0	0	4	1	0	0	0	1	0	0
Xn	0	0	0	0	0	0	0	0	0	0	0	0	0	0	0	0	1	0	1	0	0	0	0	0	0	0	1	0	1	0	28	5	3	0	0	0	0	0
