# Supplementary material for: Two decades of climate driving the dynamics of functional and taxonomic diversity of a tropical small mammal community in western Mexico
Source: PLoS One. 2017 Dec 11;12(12):e0189104. doi: 10.1371/journal.pone.0189104 (PMC5724848; doi:10.1371/journal.pone.0189104)
Supplement: S13 Table — Abbreviations are: Bm (Baiomys musculus), Lp (Liomys pictus), Mg (Megasorex gigas), Ns (Nyctomys sumichrasti), Oc (Oryzomys mexicanus), Om (Oryzomys melanotis), Ob (Osgoodomys banderanus, Pp (Peromyscus perfulvus), Rf (Reithrodontomys fulvescens), Sm (Sigmodon mascotensis), Sp (Spilogale pygmaea), Tc (Tlacuatzin canescens), Xn (Xenomys nelsoni). D (Dry season), W (wet season). (PDF) [file pone.0189104.s022.pdf]

S13 Table. Abundance database arroyo forest. Abbreviations are: Bm (*Baiomys musculus), Lp (Liomys pictus), Mg (Megasorex gigas), Ns (Nyctomys sumichrasti), Oc (Oryzomys mexicanus), Om (Oryzomys melanotis), Ob (Osgoodomys banderanus, Pp (Peromyscus perfulvus), Rf (Reithrodontomys fulvescens), Sm (Sigmodon mascotensis), Sp (Spilogale pygmaea), Tc (Tlacuatzin canescens), Xn (Xenomys nelsoni). D (Dry season), W (wet season).*

Spp	1990		1991		1992		1993		1994		1995		1996		1997		1998		1999		2000		2001		2002		2003		2004		2005		2006		2007		2012	
	D	W	D	W	D	W	D	W	D	W	D	W	D	W	D	W	D	W	D	W	D	W	D	W	D	W	D	W	D	W	D	W	D	W	D	W	D	W
Bm	0	0	0	0	0	0	0	0	6	3	0	1	0	0	0	0	0	0	0	0	0	0	0	0	0	0	0	0	0	0	0	0	0	0	3	2	0	0
Lp	129	363	118	126	36	202	0	196	62	197	68	92	96	139	51	144	118	71	51	81	215	286	80	56	76	130	62	40	130	157	188	66	36	28	73	87	18	57
Mg	0	0	0	0	0	0	0	0	0	0	0	0	0	0	1	0	0	0	0	0	0	0	0	0	0	0	0	0	1	0	0	0	0	0	0	0	0	0
Ns	6	9	29	5	0	7	18	5	7	23	4	7	3	17	9	15	7	0	1	0	2	4	2	0	1	2	1	1	5	3	0	0	3	0	0	0	0	0
Oc	33	8	5	0	0	4	0	26	40	67	15	1	0	6	39	18	25	1	16	5	18	4	11	6	0	0	10	0	71	39	1	0	0	0	5	6	17	9
Om	54	4	1	1	0	0	0	0	7	0	6	1	76	6	78	25	21	2	22	8	11	0	10	1	0	0	9	2	43	3	0	0	0	0	1	0	4	4
Ob	10	65	25	12	4	18	20	39	39	21	25	25	83	54	60	37	33	22	43	19	86	20	45	16	14	11	23	17	43	15	19	0	4	12	20	16	13	12
Pp	30	51	28	12	0	2	11	26	48	45	27	19	49	24	14	16	35	20	17	9	44	16	34	18	20	16	45	27	75	52	0	1	4	4	11	23	11	1
Rf	0	0	12	1	0	3	2	0	8	5	0	0	6	2	2	0	22	0	0	0	0	0	0	0	0	0	0	0	1	0	0	1	0	0	3	0	0	0
Sm	0	0	0	1	0	0	0	0	7	1	3	9	0	7	0	6	0	0	0	0	0	0	0	0	0	0	3	4	10	7	0	2	0	0	0	0	0	0
Sp	0	1	1	0	0	1	0	1	0	1	1	0	0	0	0	0	0	0	0	0	0	0	0	0	0	0	0	0	0	0	0	0	0	0	0	0	0	0
Tc	1	4	0	2	0	3	7	7	3	1	5	0	5	1	1	1	0	0	0	0	0	0	0	0	0	0	0	0	0	0	4	1	0	1	1	0	0	0
Xn	0	0	0	0	0	0	0	0	0	0	0	0	0	0	0	0	2	0	0	0	0	0	0	0	0	0	2	0	0	0	28	5	1	0	0	0	0	0
