# Supplementary material for: Two decades of climate driving the dynamics of functional and taxonomic diversity of a tropical small mammal community in western Mexico
Source: PLoS One. 2017 Dec 11;12(12):e0189104. doi: 10.1371/journal.pone.0189104 (PMC5724848; doi:10.1371/journal.pone.0189104)
Supplement: S14 Table — Abbreviations are: Bm (Baiomys musculus), Lp (Liomys pictus), Mg (Megasorex gigas), Ns (Nyctomys sumichrasti), Oc (Oryzomys mexicanus), Om (Oryzomys melanotis), Ob (Osgoodomys banderanus, Pp (Peromyscus perfulvus), Rf (Reithrodontomys fulvescens), Sm (Sigmodon mascotensis), Sp (Spilogale pygmaea), Tc (Tlacuatzin canescens), Xn (Xenomys nelsoni). (PDF) [file pone.0189104.s023.pdf]

S14 Table. Average weight of species. Abbreviations are: Bm (*Baiomys musculus), Lp (Liomys pictus), Mg (Megasorex gigas), Ns (Nyctomys sumichrasti), Oc (Oryzomys mexicanus), Om (Oryzomys melanotis), Ob (Osgoodomys banderanus, Pp (Peromyscus perfulvus), Rf (Reithrodontomys fulvescens), Sm (Sigmodon mascotensis), Sp (Spilogale pygmaea), Tc (Tlacuatzin canescens), Xn (Xenomys nelsoni).*

Spp	Average weight in grams
Bm	13.3
Lp	42.5
Mg	9.7
Ns	40.5
Oc	40.1
Om	32.5
Ob	37.4
Pp	37.2
Rf	15.2
Sm	82.6
Sp	162.0
Tc	27.0
Xn	105.1
